# Relationships between estimated autozygosity and complex traits in the UK Biobank

**DOI:** 10.1371/journal.pgen.1007556

**Published:** 2018-07-27

**Authors:** Emma C. Johnson, Luke M. Evans, Matthew C. Keller

**Affiliations:** 1 Department of Psychiatry, Washington University School of Medicine, St. Louis, Missouri, United States of America; 2 Institute for Behavioral Genetics, University of Colorado Boulder, Boulder, Colorado, United States of America; 3 Department of Ecology and Evolutionary Biology, University of Colorado Boulder, Boulder, Colorado, United States of America; 4 Department of Psychology and Neuroscience, University of Colorado Boulder, Boulder, Colorado, United States of America; University of Washington, UNITED STATES

## Abstract

Inbreeding increases the risk of certain Mendelian disorders in humans but may also reduce fitness through its effects on complex traits and diseases. Such inbreeding depression is thought to occur due to increased homozygosity at causal variants that are recessive with respect to fitness. Until recently it has been difficult to amass large enough sample sizes to investigate the effects of inbreeding depression on complex traits using genome-wide single nucleotide polymorphism (SNP) data in population-based samples. Further, it is difficult to infer causation in analyses that relate degree of inbreeding to complex traits because confounding variables (e.g., education) may influence both the likelihood for parents to outbreed and offspring trait values. The present study used runs of homozygosity in genome-wide SNP data in up to 400,000 individuals in the UK Biobank to estimate the proportion of the autosome that exists in autozygous tracts—stretches of the genome which are identical due to a shared common ancestor. After multiple testing corrections and controlling for possible sociodemographic confounders, we found significant relationships in the predicted direction between estimated autozygosity and three of the 26 traits we investigated: age at first sexual intercourse, fluid intelligence, and forced expiratory volume in 1 second. Our findings corroborate those of several published studies. These results may imply that these traits have been associated with Darwinian fitness over evolutionary time. However, some of the autozygosity-trait relationships were attenuated after controlling for background sociodemographic characteristics, suggesting that alternative explanations for these associations have not been eliminated. Care needs to be taken in the design and interpretation of ROH studies in order to glean reliable information about the genetic architecture and evolutionary history of complex traits.

## Author summary

Inbreeding is well known to increase the risk of rare, monogenic diseases, and there has been some evidence that it also affects complex traits, such as cognition and educational attainment. However, difficulties can arise when inferring causation in these types of analyses because of the potential for confounding variables (e.g., socioeconomic status) to bias the observed relationships between distant inbreeding and complex traits. In this investigation, we used single-nucleotide polymorphism data in a very large (N > 400,000) sample of seemingly outbred individuals to quantify the degree to which distant inbreeding is associated with 26 complex traits. We found robust evidence that distant inbreeding is inversely associated with fluid intelligence and a measure of lung function, and is positively associated with age at first sex, while other trait associations with inbreeding were attenuated after controlling for background sociodemographic characteristics. Our findings are consistent with evolutionary predictions that fluid intelligence, lung function, and age at first sex have been under selection pressures over time; however, they also suggest that confounding variables must be accounted for in order to reliably interpret results from these types of analyses.

## Introduction

Inbreeding occurs when genetic relatives have offspring, and is associated with increased risk of disorders and decreased health and viability in offspring [1–3]. This effect, called inbreeding depression, is thought to occur because natural selection more efficiently removes additive and dominant deleterious alleles, leaving the remaining deleterious alleles segregating in the population at a given time more recessive than otherwise expected [4], a phenomenon called directional dominance. Inbreeding is thought to be associated with lower fitness because it leads to long stretches of the genome that are autozygous—homozygous because the genomic segments inherited from each parent are from the same ancestor. Autozygosity reveals the full deleterious effects of recessive or partially recessive alleles that exist in these regions, and so individuals with increased autozygosity are more likely to exhibit deficits in traits that have been associated with Darwinian fitness over evolutionary time. Thus, one major reason for the interest in studying the effects of inbreeding on complex traits has been that such studies can provide insight into which traits have been under natural selection.

Because all humans are related to one another, even if distantly, inbreeding is a matter of degree. In the last decade, the increasing availability of genome-wide single nucleotide polymorphism (SNP) data has allowed scientists to infer degree of distant inbreeding, or the proportion of the genome that is autozygous, using runs of homozygosity (ROHs)—long stretches of SNPs that are homozygous [5]. The total proportion of the genome contained within these homozygous regions is called *F*_*ROH*_ and has been shown to be the best genome-wide estimate of autozygosity [5,6]. However, very large samples (e.g. *n* > 10,000) are required to detect likely effects of *F*_*ROH*_ in outbred human populations because of the low variance in levels of genome-wide autozygosity in such populations. Previous studies of *F*_*ROH*_ in humans have found evidence consistent with inbreeding depression for several complex traits, including height, forced expiratory volume in one second (FEV1), educational attainment, and cognitive ability (*g*) [7–10], with less conclusive evidence for an effect of inbreeding on psychiatric disorders [11,12] or risk factors for late-onset diseases like hypertension and other cardiovascular disease [13,14]. These observed associations with *F*_*ROH*_ may suggest that directional selection has acted on these traits ancestrally.

One challenge in autozygosity research in humans is in the causal interpretations of any observed *F*_*ROH*_ -trait relationships. It is likely that propensity to outbreed (choosing mates who are genetically dissimilar) is related to multiple sociodemographic variables in parents (e.g., education, religiosity, socioeconomic status), and these parental trait values may influence offspring trait values, thereby inducing an *F*_*ROH*_ -trait relationship that has nothing to do with the genetic effects of inbreeding depression. In a recent study conducted in the Netherlands, a relatively small, densely populated country with a strong history of latitudinal religious assortment, Abdellaoui et al. [15] found a significant association between decreased *F*_*ROH*_ (i.e. less inbred) and increased risk for major depressive disorder (MDD); this counter-intuitive association disappeared when the models accounted for religious assortment. This suggests that the original *F*_*ROH*_*−*MDD association occurred for sociological rather than genetic reasons: religious individuals had higher average levels of autozygosity than non-religious individuals, probably due to denominational restrictions on mate choice that were only recently relaxed [15], and religious individuals were less likely to experience MDD [16]. In another recent study, the largest (N > 300,000) *F*_*ROH*_ analysis to date, Joshi et al. (2016) found a significant relationship between *F*_*ROH*_ and four complex traits: height, FEV1, cognitive ability (*g*), and educational attainment [7]. When educational attainment was included as a covariate in the model as a proxy for socioeconomic status (SES), the effects for height, FEV1, and cognitive ability remained significant. Because of the persistence of these effects after accounting for educational attainment, the authors conclude that the relationship they observed between *F*_*ROH*_ and the complex traits is likely a due to a genetic mechanism, directional dominance, rather than to sociodemographic confounds. However, the *F*_*ROH*_–trait effect sizes decreased by ~20–35% after controlling for SES; it is possible that inclusion of additional, or more relevant, sociodemographic covariates could have changed these conclusions.

The findings from our work and others on the relationship between *F*_*ROH*_ and psychiatric disorders in ascertained samples [11,12,17–21] have been inconsistent and highlight concerns about the potential for unmeasured confounders to influence *F*_*ROH*_ results. Using the Psychiatric Genomics Consortium (PGC) MDD data from 9 samples, Power et al.[12] found a significant positive relationship between *F*_*ROH*_ and MDD in three German samples but, strangely, a significant negative relationship between *F*_*ROH*_ and MDD in six samples from non-German sites. Similarly, in 2012 we found a small but highly significant association between schizophrenia and *F*_*ROH*_ across 17 case-control datasets (total N = 21,844 [20]). However, in 2016 we published an independent replication using the same procedures as our previous study that found little to no evidence of an *F*_*ROH*_-schizophrenia association across 22 case-control datasets (total N = 39,830 [11]). We are uncertain how to explain these discrepancies, but we have hypothesized that unmeasured cofounding variables such as education, religiosity, and income can differentially bias such ROH findings across different sites, and that this problem is particularly salient in ascertained samples where cases and controls may be drawn from subpopulations that differ slightly on background sociodemographic characteristics. While such differences in ascertainment between cases and controls are unlikely to lead to significant allele frequency differences, and thus are unlikely to bias genome-wide association studies (GWAS), they could easily lead to systematic case-control differences in *F*_*ROH*_, depending on the difference in degree of inbreeding in the subpopulations from which cases and controls were drawn.

Finally, a recent study by Yengo et al. [10] quantified a separate source of potential bias in *F*_*ROH*_ analyses. Using simulations, the authors show that *F*_*ROH*_ -based measures of inbreeding can overestimate the inbreeding effect size compared to SNP-based measures. The authors go on to show that a SNP-based measure of inbreeding calculated from the correlation between uniting gametes, *F*_*UNI*_, provides unbiased estimates of inbreeding effects when causal variants are well-tagged by measured SNPs. However, unlike *F*_*ROH*_, which captures the effects of both common and rare recessive causal variants [5], SNP-based measures of inbreeding such as *F*_*UNI*_ underestimate the effects of (typically rare) recessive causal variants that are poorly tagged by measured SNPs [22]. We chose to focus on *F*_*ROH*_ rather than SNP-based measures of inbreeding in the current study because we were interested in investigating the evidence for directional dominance on complex traits, which is theoretically manifested via a higher proportion of rare and recessive causal variants. We also chose to focus on *F*_*ROH*_ for comparability with previous findings, as *F*_*ROH*_ has been the standard metric in the field for assessing inbreeding depression to date.

Here, we describe the most powerful investigation to date of the association of *F*_*ROH*_ with several complex traits. We used whole-genome SNP and phenotypic data from the UK Biobank (total *n* ~ 100,000–400,000) to address two principal questions: (1) is there evidence consistent with directional dominance on traits related to fitness and health, such that increased *F*_*ROH*_ is associated with lower trait values? and (2) do *F*_*ROH*_-trait relationships persist after controlling for multiple background sociodemographic variables? This sample is population-based, reducing concerns about ascertainment-induced confounds, and includes information on multiple relevant sociodemographic control variables and traits previously associated with *F*_*ROH*_ (e.g. waist-to-hip ratio, grip strength, diastolic and systolic blood pressure, and fluid intelligence [7–10,12–14]), making it an ideal sample for investigating the relationship between distant inbreeding and complex traits.

## Methods

### Ethics statement

This study utilized de-identified data from the UK Biobank. UK Biobank received ethical approval from the NHS National Research Ethics Service North West (11/NW/0382).

### UK Biobank sample

Our data came from the UK Biobank, a population-based sample from the United Kingdom. In total, 502,682 individuals were recruited from 2006–2010 from 22 centers across the UK. Participants were given a touchscreen interview that included questions about demographic characteristics, health history, and lifestyle information (e.g. diet, alcohol intake, sleep habits), and some anthropometric and physical measures were collected. DNA was extracted from whole blood and genotyped using either the Affymetrix UK Biobank Axiom array or the Affymetrix UK BiLEVE Axiom array. Detailed genotyping and sample QC procedures are described in Bycroft et al. [23] We analyzed data from the second release of up to 400,000 individuals (exact *N* varied by phenotype) with genotypes available.

### Phenotypes

We examined 26 traits related to health, fitness, or sociodemographic characteristics (see S1 Text for full description and field ID of individual measures). These included 17 continuous traits (age at first sexual intercourse, waist to hip ratio, height, body mass index (BMI), basal metabolic rate (BMR), diastolic and systolic blood pressure (BP), hand-grip strength (taking the maximum of left and right grip strength measurements), county-wide socioeconomic status (SES) as measured by the Townsend Deprivation Index (TDI), total household income (an ordinal variable of income brackets recoded to be numeric, ranging from 0–4), years of educational attainment (coded using ISCED classifications as in Okbay et al. [24]), fluid intelligence score (FI), forced expiratory volume in 1 second (FEV1; a measure of lung function), FEV1 over forced vital capacity (FEV1/FVC), birth weight, neuroticism score, and body fat percentage) and 9 binary traits (ever smoked, ever drank alcohol, whether or not they were breastfed as a baby, whether or not they completed college, whether they specified participation in a religious group as a leisure activity, whether or not they had ever been diagnosed with diabetes, probable bipolar and/or major depression status, and whether they live in an urban or rural area). These traits were chosen because they had either been previously studied in ROH analyses (fluid intelligence, grip strength, FEV1, FEV1/FVC ratio, waist-to-hip ratio, height, BMI, diastolic and systolic blood pressure [7–10]), were diagnoses or binary traits of psychiatric and biomedical interest with large enough Ns to reasonably include in our study (diabetes, probable bipolar or major depressive disorder diagnoses, ever smoked, ever drank), or were our only available proxy measure of reproductive success (age at first sexual intercourse). Our “sociodemographic” traits were chosen because of their hypothesized (regional poverty via Townsend Deprivation Index [25], breastfed as an infant as a proxy for mother’s socioeconomic status [26], income, urbanicity) or previously demonstrated (education, religious assortment [15,27]) influence on ROH–trait associations. We excluded individuals who weighed less than 36.28 kg (~80 lbs), weighed more than 6.8 kg (~15 lbs) at birth, had systolic BP readings >200 mmHg or diastolic BP readings >120 mmHg, were shorter than 120 cm (~3.93 ft), had a hip circumference <50 cm or >175 cm, had a waist circumference <40 cm or >160 cm, had grip strength >70 kg, or reported having had sex before 12 years of age. These exclusion criteria were chosen based on thresholds typically defined as being boundaries of normal physiological, anthropometric, or behavioral ranges and by checking for obvious outliers that may have been incorrect data entries. More information on specific phenotype derivations and calculations are included in the supplemental material. We standardized all quantitative phenotypes (within sex) before calculating their relationship with *F*_*ROH*_ for ease of comparison with Joshi et al.’s and others’ results [7].

### Quality control (QC)

Because the sample was predominately European ancestry, we restricted analyses to individuals of European ancestry (N = 436,065) as identified by visual inspection of plots of genomic principal components. We followed sample and genotypic quality control that has become typical in ROH analyses. In particular, we excluded SNPs if they a) deviated from Hardy-Weinberg equilibrium at p<1×10^−6^, b) missing call rate > 0.02, or c) had a minor allele frequency (MAF) < 0.05. We also excluded individuals with discordant self-reported gender and genetic sex, a missing genotype call rate > 0.02, and we removed the minimum number of individuals so that all remaining subjects were unrelated at $\hat{\pi }$ > 0.2 (using GCTA’s—grm-cutoff option [28]) (n = 31,541 removed in total). We also repeated these analyses after using a stricter relatedness cutoff, removing all individuals related at $\hat{\pi }$ > 0.05 (n = 103,389 removed in total; see S1 Text), to ensure the robustness of our results.

### ROH calling procedures

After QC, we pruned out SNPs that were in strong linkage disequilibrium with other SNPs by removing those that had a variance inflation factor > 10 (equivalent to an r^2^ of 0.90) between target SNPs and 50 surrounding SNPs (plink command:—indep 50 5 10). After these procedures, 263,609 SNPs and 404,524 individuals remained. For our main analysis, we called ROHs as being ≥65 homozygous SNPs in a row, set the minimum KB length very low (essentially ignoring the length requirements), with no heterozygote calls and three missing variant calls allowed (5% of the SNP threshold), per recommendations from Howrigan et al. (2011) for genotype data of similar SNP density. We required ROHs to have a density greater than at least 1 SNP per 200 kb (the average density across the genome in the SNPs used in the analysis was 1 per 10 kb) and split an ROH into two if a gap >500 kb existed between consecutive homozygous SNPs. These analyses used the following commands in Plink 1.9 [29]:—homozyg-window-snp 65—homozyg-snp 65—homozyg-kb 10—homozyg-gap 500—homozyg-window-missing 3—homozyg-window-het 0—homozyg-density 200 (see S1 Text for further discussion of parameter choice). After calling ROHs, we summed the total length of all autosomal ROHs for each individual and divided that by the total SNP-mappable distance (2.77x10^9^ bases) to calculate *F*_*ROH*_, the proportion of the genome likely to be autozygous.

We also tested the relative importance of recent vs. distant inbreeding by calculating *F*_*ROH*_ from longer ROHs (indicative of closer inbreeding) and comparing to the effect of *F*_*ROH*_ from shorter ROHs (a proxy for more distant inbreeding). We defined recent inbreeding as the proportion of the genome contained in autozygous regions longer than 8.5 Mb (*F*_*ROH_long*_) and distant inbreeding as the proportion of the genome in autozygous regions shorter than 8.5 Mb (*F*_*ROH_short*_), as *F*_*ROH_long*_ and *F*_*ROH_short*_ had approximately equal variances (4.5e-6 and 4.3e-6, respectively) in our sample. An autozygous segment spanning < 8.5 Mb should originate from a common ancestor at least 6 generations ago on average [30].

In addition to calling ROHs, we also calculated a measure of SNP-by-SNP homozygosity (*F*_*SNP*_) for each individual, using the—het flag in Plink 1.9 [29]:

*F*_*SNP*_ = [observed homozygous count—expected count] / [total observations—expected count]

Because it is calculated with genotyped SNPs, *F*_*SNP*_ is a measure of excess homozygosity at common SNPs (see S3 Table for the correlation matrix between *F*_*SNP*_, *F*_*ROH_long*_ and *F*_*ROH_short*_).

### ROH burden analysis

*F*_*ROH*_ was used as the primary predictor of the traits of interest in analyses described below. The distributions of ROH lengths and *F*_*ROH*_ are shown in S1 Fig. We regressed each trait (Y) on *F*_*ROH*_ using the model in the equation below, where ${\hat{\beta }}_{0}$ is the intercept, ***C*** is a matrix of covariates (including e.g. the first 20 principal components) and ε represents the residual error term.

$$\vec{Y}={\hat{\beta }}_{0}+{\hat{\beta }}_{1}F_{ROH}+\vec{\gamma }C+\varepsilon$$

As noted above, all quantitative phenotypes were standardized to intra-sex *z-*scores for ease of comparison with previous findings in the literature. In addition, for ease of interpretation, we reverse-coded some of the phenotypes such that lower values represented what we thought were likely to be lower fitness and/or less desirable outcomes (e.g. disease diagnosis was coded as ‘0’ while no diagnosis was coded as ‘1’, and TDI was reverse-coded such that lower values represented greater material poverty). We were primarily interested in the estimate of ${\hat{\beta }}_{1}$, which represents the association of *F*_*ROH*_ with the trait, controlling for covariates (although in one set of models, described below, we were also interested in the effect of *F*_*SNP*_ on the trait). For binary traits, we ran logistic regression models with the same covariates as in the linear regression models for quantitative traits.

We ran a total of three sets of models for each trait. The first set of models was designed to test for a simple relationship between *F*_*ROH*_ and the traits listed above. Because confounding factors such as population stratification, SNP missingness, call quality, and plate effects can influence *F*_*ROH*_, we included the batch number, percentage of missing SNP calls per sample in the non-imputed genotype data, and the first 20 ancestry principal components (calculated within individuals of European ancestry), as well as age, age^2^, and sex, in all of the regression models unless explicitly stated.

In our second set of models, we tested whether background sociodemographic characteristics mediated *F*_*ROH*_-trait relationships. In addition to the above covariates, in these models we also included income, years of educational attainment, Townsend Deprivation Index (a measure of the amount of material deprivation in a given region [25]), and whether subjects attended college, lived in an urban area, participated in a religious group as a leisure activity, and reported being breastfed as an infant. Although the covariates of true interest are those measured on the parents (whose sociodemographic traits may influence mate choice), parental information was unavailable (other than breastfeeding, which is associated with mother’s socioeconomic status [26]), and so we used the subjects’ own values on these traits as the best available proxies for characteristics of their parents.

In our third set of models, we tested the degree to which observed *F*_*ROH*_-trait relationships were due to homozygosity at common versus rare alleles. To do this, we included *F*_*SNP*_ as a covariate in addition to the covariates from the second set of models above. Because common SNPs can often predict (are in linkage disequilibrium with) other common SNPs but typically poorly predict rare SNPs, *F*_*SNP*_ captures effects of homozygosity at common SNPs only whereas *F*_*ROH*_ captures the effects of homozygosity at both common and rare SNPs [5]. In the S1 Text (and S4 Table), we demonstrate via simulation that entering both *F*_*SNP*_ and *F*_*ROH*_ as predictors simultaneously in the regression equation allows insight into the degree to which observed inbreeding effects are due to homozygosity at common versus rare alleles.

## Results

**Fig 1:**
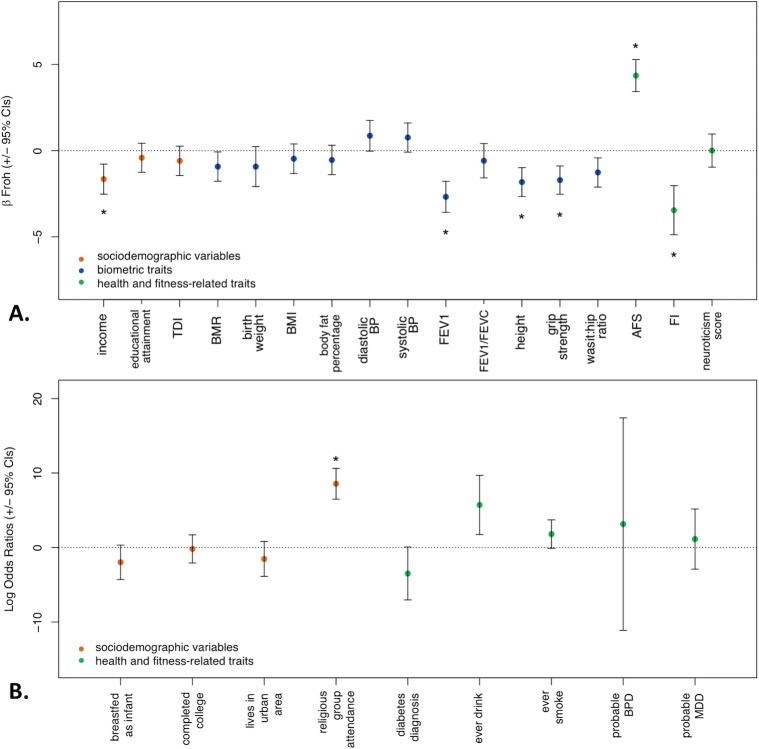
Beta *F*_*ROH*_ and 95% confidence intervals from main regression models controlling for minimal covariates (20 ancestry principal components, genotype batch, per-sample SNP missingness, age, age^2^, and sex). Significant estimates (at p < 0.002—corrected for multiple testing) are starred (religious group attendance as a leisure activity, income, AFS, FEV1, FI, height, and grip strength). **A.** All quantitative traits were analyzed in intra-sex standardized phenotypic units in linear regression models. **B.** Binary traits and diagnoses were analyzed using logistic regression models (the log odds ratios are reported). AFS, age at first sexual intercourse; BMI, body mass index; BMR, basal metabolic rate; BP, blood pressure; BPD, bipolar disorder; CI, confidence interval; FEV1, forced expiratory volume in 1 second; FI, fluid intelligence; FVC, forced vital capacity; MDD, major depression; TDI, Townsend Deprivation Index.

The distribution of ROH lengths, *F*_*ROH*_, and *F*_*SNP*_ are shown in S1 and S2 Figs, and descriptive statistics are given in S1 Table. Using a Bonferroni correction based on testing 26 traits (α = 0.002), we observed significant negative associations between *F*_*ROH*_ and income, grip strength, height, fluid intelligence score (FI), and forced expiratory volume in one second (FEV1), and observed significant positive associations between *F*_*ROH*_ and age at first sexual intercourse (AFS) and religious group participation (Table 1 and Fig 1). The associations we found between *F*_*ROH*_ and FI, FEV1, and height replicate three of Joshi et al.’s four significant findings. To our surprise, we did not replicate their significant relationship between *F*_*ROH*_ and educational attainment. When these analyses were repeated in the smaller sample of individuals unrelated at $\hat{\pi }$ > 0.05, conclusions did not change (see S5 Table). When we tested the effects of recent vs. distant inbreeding, the results for more recent inbreeding were similar to the full *F*_*ROH*_ models: income, grip strength, height, FI, FEV1, AFS, and religious group participation were all associated with *F*_*ROH_long*_, with the same direction of effect as the original models. Similarly, AFS, FEV1, FI, religious group attendance, and ever drink (such that being more autozygous was associated with a lower likelihood of having ever drank alcohol) were significantly associated with *F*_*ROH_short*_, while its associations with income, grip strength, and height were not significant (S6 Table).

**Table 1:** **Association of *F***_***ROH***_ **with 26 traits, in two sets of models: 1) controlling for age, age**^**2**^**, sex, the first 20 principal components, sample missingness, and batch number as covariates, and 2) also controlling for sociodemographic variables**.

			Main models—controlling for batch, sample missingness, sex, age, age^2^, and first 20 principle components			Models also controlling for sociodemographic covariates (income, educational attainment, college, urban, TDI, religiosity, whether or not breastfed)		
Category	Trait	N	Beta	SE	p	Beta	SE	p
**Quantitative Traits (linear regression)**								
Sociodemographic	**Income**	347883	-1.648	0.446	2.18E-04			
Sociodemographic	years of education	400383	-0.410	0.429	0.340			
Sociodemographic	Townsend Deprivation Index	404034	-0.590	0.434	0.173			
Biometric	basal metabolic rate	397363	-0.922	0.434	0.034	-0.825	0.568	0.146
Biometric	birth weight	229569	-0.925	0.589	0.116	-0.966	0.661	0.144
Biometric	body mass index	403173	-0.469	0.437	0.284	-0.150	0.562	0.789
Biometric	body fat percentage	397148	-0.539	0.436	0.216	-0.129	0.563	0.819
Biometric	diastolic BP	380686	0.864	0.452	0.056	1.218	0.585	0.037
Biometric	systolic BP	379733	0.763	0.432	0.077	0.398	0.551	0.470
Biometric	forced expiratory volume in 1 second (FEV1)*	304301	-2.677	0.458	5.20E-09	-2.791	0.580	1.51E-06
Biometric	FEV1/FVC	304301	-0.584	0.508	0.250	0.318	0.637	0.617
Biometric	**height**	403609	-1.821	0.427	1.99E-05	-1.150	0.548	0.036
Biometric	**grip strength**	403589	-1.706	0.420	4.81E-05	-1.368	0.541	0.012
Biometric	waist to hip ratio	403689	-1.262	0.431	0.003	-1.443	0.551	0.009
Health- and fitness-related	age at first sexual intercourse*	354311	4.355	0.474	3.97E-20	3.479	0.569	9.73E-10
Health- and fitness-related	fluid intelligence*	145658	-3.455	0.725	1.90E-06	-3.414	0.847	5.58E-05
Health- and fitness-related	neuroticism score	327994	0.008	0.490	0.987	0.403	0.614	0.512
**Binary Outcomes (logistic regression)**								
Sociodemographic	breastfed as infant	305904	-1.974	1.177	0.093			
Sociodemographic	college degree	404518	-0.184	0.963	0.848			
Sociodemographic	live in urban area	400629	-1.525	1.190	0.200			
Sociodemographic	**religious group attendance**	404518	8.568	1.056	5.00E-16			
Health- and fitness-related	diagnosed with diabetes	403387	-3.486	1.808	0.054	-3.580	2.406	0.137
Health- and fitness-related	ever drink	403990	5.720	2.026	0.005	2.840	2.950	0.336
Health- and fitness-related	ever smoke	365395	1.805	0.970	0.063	0.230	1.270	0.856
Health- and fitness-related	Probable BPD diagnosis	71007	3.145	7.273	0.665	4.746	8.312	0.568
Health- and fitness-related	Probable MDD diagnosis	95481	1.134	2.057	0.581	-0.653	2.501	0.794

* Phenotypes significantly associated with *F*_*ROH*_ after controlling for sociodemographic covariates.

Betas are reported for all quantitative traits, which were analyzed in within-sex standardized phenotypic units; the betas reported for binary traits and diagnoses are log odds ratios, as these outcomes were analyzed using logistic regression models. Phenotypes with a significant relationship with *F*_*ROH*_ (*p* < 0.002 after multiple testing correction) are bolded; those with an asterisk are also significantly associated with *F*_*ROH*_ after controlling for sociodemographic covariates (income, educational attainment, college degree, urbanicity, TDI, religious group participation, and whether or not they were breastfed as an infant). Reported N is the number of individuals with non-missing information for the outcome trait. Sociodemographic variables are listed first, followed by biometric measures, with health- and fitness-related traits listed last. BP, blood pressure; FVC, forced vital capacity; BPD, bipolar disorder; MDD, major depressive disorder; df, degrees of freedom; OR, odds ratio; SE, standard error.

**Fig 2:**
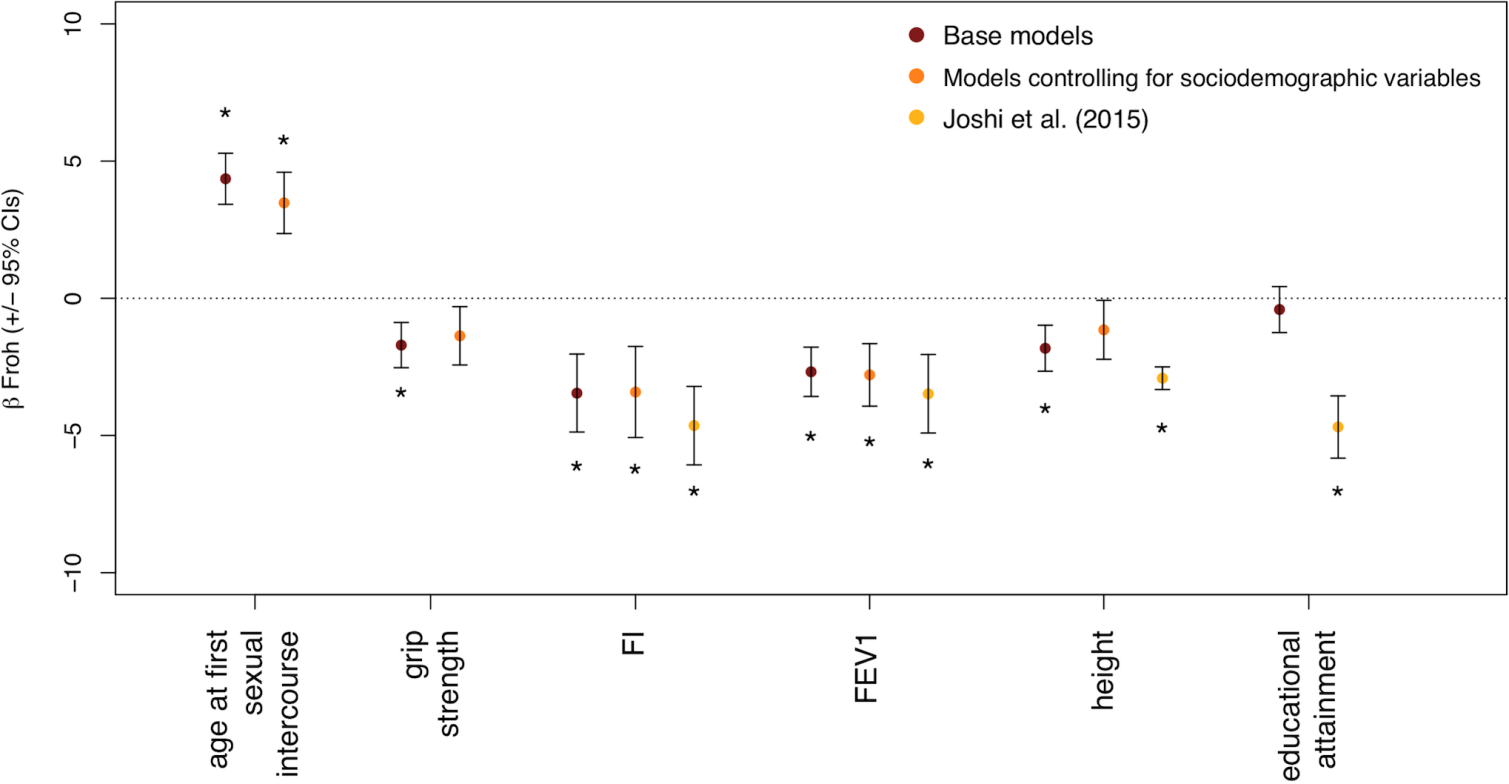
Comparison with estimates from Joshi et al. 2015, and some evidence that sociodemographic background variables attenuate the relationship between *F*_*ROH*_ and complex traits. Plot shows the Beta *F*_*ROH*_ and 95% confidence interval in within-sex standardized phenotypic units for the five quantitative traits that were significantly associated with *F*_*ROH*_ in the main models (Fig 1), as well as educational attainment, which was significantly associated with autozygosity in Joshi et al.’s study^7^. Estimates that were statistically significant after multiple testing corrections are starred for each set of models. After controlling for background sociodemographic characteristics, AFS, FEV1, and FI were still statistically significant in our study. The effect sizes for AFS, grip strength, FI, and height all decreased after controlling for sociodemographic variables. The effect sizes from our analyses were smaller for all four of the phenotypes also measured in Joshi et al.’s study. FI, fluid intelligence; FEV1, forced expiratory volume in 1 second; CI, confidence interval.

When we included the seven sociodemographic variables as covariates in the regression models (other than those predicting sociodemographic variables), the betas associated with *F*_*ROH*_ decreased for AFS, grip strength, height, and FI (by 20.1%, 19.8%, 36.8%, and 1.2%, respectively) and increased for FEV1 (by 4.2%) (see Table 1 and Fig 2). AFS, FI, and FEV1 remained significantly associated with *F*_*ROH*_ whereas the associations with height and grip strength became non-significant. No significant indirect mediation effect of the sociodemographic variables in combination was found for the relationships between *F*_*ROH*_ and AFS, grip strength, height, FI, or FEV1 (see S1 Text for a description of these tests). Furthermore, the association between *F*_*ROH_short*_ and ever drink became non-significant after controlling for the sociodemographic covariates, as did the associations between *F*_*ROH_long*_ and grip strength, height, and FI (S8 Table). Finally, we tested whether the effect of *F*_*ROH*_ differed by sex by including sex*F_*ROH*_ interaction terms in each of the second set of models; we observed no significant sex-by-*F*_*ROH*_ interactions for any of the traits.

In our final set of models, where excess SNP-by-SNP homozygosity (*F*_*SNP*_) was included as an additional covariate, AFS and FI remained significantly associated with *F*_*ROH*_ after accounting for multiple testing and FEV1 was marginally significant (Table 2). Waist-to-hip ratio was significantly associated with *F*_*SNP*_ but not *F*_*ROH*_, suggesting that higher homozygosity at common but not rare variants is related to increased waist-to-hip ratio. When these analyses were repeated in the smaller set of individuals unrelated at a stricter cutoff ($\hat{\pi }$ < 0.05), our findings did not substantially change, though fluid intelligence no longer met the *p*-value cutoff for determining statistical significance, likely because of larger standard errors from the reduced sample size (S9 Table).

**Table 2:** Effects of both *F*_*ROH*_ and excess SNP-by-SNP homozygosity, measured by *F*_*SNP*_, controlling for the same covariates as in the previous models (age, age^2^, sex, batch number, per-sample SNP missingness, the first 20 principal components, and background sociodemographic variables.).

			*F*_*ROH*_ *Regression Coefficient*			*F*_*SNP*_ *Regression Coefficient*		
Category	Trait	N	Beta_*FROH*_	SE_*FROH*_	*p*_*FROH*_	Beta_*FSNP*_	SE_*FSNP*_	*p*_*FSNP*_
	**Quantitative Traits (linear regression)**							
Biometric	basal metabolic rate	397363	-0.883	0.678	0.193	0.056	0.361	0.876
Biometric	birth weight	229569	-1.443	0.792	0.069	0.465	0.426	0.275
Biometric	body mass index	403173	-0.230	0.672	0.732	0.078	0.358	0.828
Biometric	body fat percentage	397148	-0.353	0.672	0.599	0.219	0.357	0.540
Biometric	diastolic BP	380686	1.334	0.700	0.057	-0.113	0.373	0.762
Biometric	systolic BP	379733	0.725	0.658	0.271	-0.318	0.351	0.365
Biometric	forced expiratory volume in 1 second (FEV1)	304301	-2.010	0.688	0.004	-0.762	0.361	0.035
Biometric	FEV1/FVC	304301	-0.110	0.755	0.884	0.418	0.396	0.291
Biometric	height	403609	-0.950	0.655	0.147	-0.195	0.349	0.576
Biometric	grip strength	403589	-0.620	0.645	0.337	-0.728	0.342	0.033
Biometric	*waist to hip ratio*	403689	-0.137	0.658	0.835	-1.273	0.351	2.88E-04
Health- and fitness-related	**age at first sexual intercourse**	354311	2.858	0.676	2.35E-05	0.602	0.354	0.089
Health- and fitness-related	**fluid intelligence**	145658	-3.238	1.016	0.001	-0.171	0.544	0.753
Health- and fitness-related	neuroticism score	327994	0.432	0.732	0.555	-0.029	0.389	0.941
	**Binary Outcomes (logistic regression)**							
Health- and fitness-related	diagnosed with diabetes	403387	-3.840	3.051	0.208	0.252	1.812	0.890
Health- and fitness-related	ever drink	403990	2.304	3.749	0.539	0.520	2.241	0.817
Health- and fitness-related	ever smoke	365395	-1.699	1.510	0.261	1.881	0.796	0.018
Health- and fitness-related	Probable BPD diagnosis	71007	7.619	10.645	0.474	-2.864	6.632	0.666
Health- and fitness-related	Probable MDD diagnosis	95481	-0.877	3.028	0.772	0.220	1.679	0.896

Phenotypes with a significant relationship with *F*_*ROH*_ (*p* < 0.003 after multiple testing correction) are bolded, while those with a significant relationship with *F*_*SNP*_ are italicized. The quantitative traits (analyzed via linear regression) are listed first in the table, followed by diagnoses and binary traits. Reported N is the number of individuals with non-missing information for the outcome trait. BP, blood pressure; FVC, forced vital capacity; BPD, bipolar disorder; MDD, major depressive disorder; df, degrees of freedom; SE, standard error.

## Discussion

### Overview of findings

We replicated several previous associations between *F*_*ROH*_ and fitness-related traits, identified a novel association between *F*_*ROH*_ and a reproductive phenotype (age at first sexual intercourse), and found weak evidence that background sociodemographic characteristics may be partially mediating a few of the observed relationships between *F*_*ROH*_ and complex traits (Fig 2). In particular, we found robust evidence that fluid intelligence (FI), forced expiratory volume in one second (FEV1), and age at first sexual intercourse (AFS) are associated with *F*_*ROH*_ (Tables 1 and 2), while grip strength and height’s relationships with *F*_*ROH*_ were attenuated enough to become non-significant after controlling for background sociodemographic variables. The associations of *F*_*ROH*_ with FI and FEV1 were especially robust, with the inclusion of sociodemographic covariates having little to no consequence on these effect sizes. The strength of *F*_*ROH*_ associations for more recent inbreeding was similar or stronger than those for more distant inbreeding, except, interestingly, for AFS, FI, and participation in a religious group. When we accounted for SNP-by-SNP homozygosity in the model, AFS and FI were still significantly associated with *F*_*ROH*_, consistent with their relationships with *F*_*ROH*_ being more strongly driven by homozygosity at rare rather than common variants. Certain other associations were likely due to social rather than genetic causes; for example, it is much more plausible that non-religious individuals tend to outbreed at higher rates and have less religious offspring than that autozygosity causes individuals to be more religious.

### Comparison with previous results

Our results largely agree with recent reports [7–10] on the relationships between estimated autozygosity and complex traits in population-based samples. Replicating Howrigan et al. [8], Joshi et al. [7], and Yengo et al.[10], as well as previous pedigree studies [31], we found a significant, negative relationship between *F*_*ROH*_ and fluid intelligence. In addition, we replicated Joshi et al.’s [7] finding of a significant relationship between increased *F*_*ROH*_ and decreased FEV1. We initially observed a significant association between increased *F*_*ROH*_ and decreased height, as did Joshi et al. [7] and Verweij et al. [9], but this association was attenuated in our sample after controlling for background sociodemographic variables and did not meet statistical significance after Bonferroni corrections (Table 1 and Fig 2). Our initial results (Table 1) were consistent with previous findings for an effect of inbreeding depression on grip strength [9], though this association appears to be more likely due to homozygosity at common rather than rare variants (Table 2).

Despite the general consistency across reports on *F*_*ROH*_-complex trait associations, there were two differences between our results and those from earlier studies. First, educational attainment (in years of education) was not significantly associated with *F*_*ROH*_ in any of our models, contrary to several previous reports [7,9], but consistent with Yengo et al. [10]. We found a significant (*p* = 2.18e-4) relationship between *F*_*ROH*_ and income (which itself was correlated with years of education at *r* = 0.37), but we found no evidence for an association between *F*_*ROH*_ and either years of education or the binary variable measuring whether or not an individual attended college. The reason for the discrepancy in findings for education is unlikely to be due to sampling variability because the two confidence intervals do not overlap (Fig 2). One possibility is that educational attainment is less correlated with geographic mobility (and the tendency to outbreed) in the UK compared to other countries previously investigated, and Joshi et al. [7] did report significant heterogeneity of the *F*_*ROH*_-education association across sites. Moreover, of the 5 cohorts from the UK investigated by Joshi et al. [7], two (GRAPHIC and LBC1936) showed associations in the opposite direction of the overall association (see their Extended Data Fig 2). Thus, it is possible that the *F*_*ROH*_ -educational attainment relationship might be different in the UK than is typical in other societies. Furthermore, the association we found between height and autozygosity was attenuated (by ~37%) when we accounted for sociodemographic covariates, and was somewhat smaller than that found by previous studies even when we did not control for sociodemographic variables (e.g. a 1% increase in *F*_*ROH*_ predicted a decrease of ~.03 s.d. of height in previous studies [7,32] versus a decrease of ~.02 s.d. in the current study). Nevertheless, the confidence intervals for Joshi et al.’s [7] and our observed association between height and *F*_*ROH*_ overlapped (Fig 2), suggesting that sampling variability could be a reason for the discrepant height findings.

In comparing results across recent publications and the current one, it is important to note the differences in populations, samples, and measurements across studies. Both Howrigan et al. [8] and Joshi et al. [7] took a meta-analytic approach, conducting *F*_*ROH*_ analyses in each contributing sample separately, and then combining across samples, controlling for relevant covariates (e.g. dataset, country of data collection). Joshi et al. in particular analyzed a much more diverse overall sample than the present study, including multiple cohorts from European, African, and Asian populations. Another difference is in the measurement of intelligence across studies: our measurement for general cognitive ability was the unweighted sum of the number of 13 fluid intelligence questions answered correctly, given as part of the UK Biobank’s cognitive function assessment, while Howrigan et al. [8] converted the scores from each contributing sample’s measure of general cognitive ability (e.g. WAIS-R, Cattell Culture Fair Test) into z-scores (to avoid bias from different measurement schemes across samples), and Joshi et al. used *g* as their measure of general cognitive ability, “calculated as the first unrotated principal component of test scores across diverse domains of cognition”. Furthermore, our regression models controlled for the first 20 ancestry principal components, while Howrigan et al. controlled for the first 10 and Joshi et al. the first 3.

### Possible evolutionary interpretations

There are two major evolutionary theories for why inbreeding depression occurs [4]: the overdominance hypothesis posits that an overall loss of heterozygosity at loci governed by heterozygote advantage leads to inbreeding depression, while the partial dominance theory postulates that inbreeding depression occurs as selection acts most efficiently on the most additive and dominant deleterious mutations, purging those from the population while leaving behind the more rare, partially recessive deleterious alleles. This second hypothesis, partial dominance, is widely accepted as the more likely mechanism of inbreeding depression [3,33]. The robust associations we observed between *F*_*ROH*_ and AFS, FI, and FEV1, even after controlling for homozygosity at common variants with *F*_*SNP*_, suggest that the variants contributing to lower trait values are biased toward being rare and recessive, consistent with predictions from a partial dominance model of inbreeding depression [5] and consistent with the hypothesis that these traits, or traits genetically correlated with them, have been under directional selection over evolutionary time. Cognitive ability, including intelligence test scores, is a predictor of multiple Darwinian fitness-related outcomes, including overall health and lifespan [8,34]. FEV1 is correlated with mortality and lifespan [35–38], traits that are components of fitness and thus more likely to have been under directional selection over evolutionary history [39]. Thus, our replication of the associations between autozygosity and FEV1 and FI adds to a body of evidence that these traits, or traits genetically correlated with them, have been under directional selection over evolutionary history, consistent with the expectation that variants that influence them are biased toward being rarer and more recessive than expected under a neutral drift model.

The positive relationship we observed between AFS and *F*_*ROH*_ is a novel finding, to the best of our knowledge, though associations between *F*_*ROH*_ and reproductive phenotypes have been observed previously, for different proxy measures (e.g. number of children fathered, in Yengo et al. [10]). The *F*_*ROH*_-AFS association was attenuated but remained statistically significant after controlling for sociodemographic variables and homozygosity at common variants (*F*_*SNP*_). Our finding is consistent with a body of research suggesting that reproductive traits, like AFS, in non-human populations are under more intense selection pressures than non-fitness traits [5,40]. If autozygosity causally influences AFS (see “Limitations” below), there are two possible evolutionary interpretations. First, it is possible that early sex itself was advantageous in ancient human history due to a prolonged reproductive period. A second possibility is that the observed association between autozygosity and AFS is due to selection on a genetically correlated trait, such as sexual attractiveness [41,42]. However, it is important to note that the original effect size for the association between *F*_*ROH*_ and AFS decreased by ~20% after accounting for sociodemographic variables. Furthermore, sociodemographic variables were not measured on parents (the more relevant control; see below), and the single dichotomous variable for religious group participation that we used as a proxy for religiosity is unlikely to capture the full confounding effects of religious observance on sexual behaviors. Thus, the association between inbreeding and age at first sexual intercourse that we observed in this study should be interpreted with caution.

### Limitations

There were three central limitations in the current study. The most important one, which applies equally to all other *F*_*ROH*_ studies that we are aware of, is that ROH associations might be due to third-variable explanations. Unlike GWAS analyses, where parental or offspring sociodemographic traits are unlikely to be associated with allele frequencies and therefore are unlikely to bias GWAS results, it takes only a single generation of parental inbreeding to strongly influence *F*_*ROH*_ levels in offspring. For example, higher income might be associated with greater opportunities to meet mates of diverse origins and to higher outbreeding; offspring of higher income parents might thereby have not only lower levels of autozygosity, on average, but might also differ on any traits influenced genetically or environmentally by parental income. While sociodemographic confounding is particularly problematic in ascertained samples where cases and controls are drawn from different populations (e.g. cases drawn from a psychiatric hospital, controls from a nearby university), the possibility of confounding cannot be eliminated, even in population-based samples, unless relevant sociodemographic variables among parents are measured and controlled for or other (e.g., within-family) designs are used. For example, in a study of approximately 2,000 individuals of Dutch ancestry, Abdellaoui et al. [27] found only a weak association between *F*_*ROH*_ and the subjects’ own educational attainment (*p* = 0.045), but found highly significant negative associations between the subject’s *F*_*ROH*_ and their parents’ educational attainment (*p*_*father*_ *<* 10^−5^, *p*_*mother*_ = 9e^-5^). These relationships were entirely mediated by the geographic distance between parents’ birthplaces, such that parents with higher educational attainment tended to be more geographically mobile, increasing their chances of mating with someone genetically dissimilar from themselves and thus creating systematic differences in levels of inbreeding across levels of educational attainment in their offspring.

Having information on parents’ birth location, education, income, mobility, level of religious involvement, and so forth is important in order to control for the possibility that these sociodemographic variables are associated with both higher levels of (distant) inbreeding and lower offspring trait values. Unfortunately, the UK Biobank has limited parental information other than indirect measures such as whether one was breastfed. In the current study, we used sociodemographic responses of the offspring as imperfect proxies for parental responses, which is effective only to the degree that offspring values on these sociodemographic variables are positively correlated with their parents’ values. For example, parental educational (*r* = 0.25–0.40; [43,44]), income (r = .60; [44]), and religiosity [45] are imperfectly correlated between parents and offspring in Great Britain. These imperfect correlations imply that the true mediating influences of the sociodemographic variables on observed *F*_*ROH*_ -trait relationships were likely to be underestimated in the present report, and thus causal interpretation of our results may not be warranted.

Still, while *F*_*ROH_short*_ is an imperfect measure of truly distant inbreeding (as recent inbreeding can also produce short ROHs), it may be that *F*_*ROH_short*_ is less susceptible to confounding from recent assortative mating than *F*_*ROH_long*_ (a measure of more recent inbreeding). The three traits (AFS, FI, and FEV1) that were significantly associated with *F*_*ROH*_ even after the inclusion of sociodemographic covariates were also significantly associated with *F*_*ROH_short*_ (S6 and S8 Tables). This bolsters our hypothesis that autozygosity is causally influencing these three traits through inbreeding depression, while the evidence for height and grip strength (which were associated with recent but not distant inbreeding, S6 Table) is weaker.

A second limitation is the potential for a collider bias in the models in which we controlled for sociodemographic variables. Collider bias occurs when a covariate in a linear model is causally influenced by both the outcome and the predictor of interest, which creates a bias in the estimated association between the predictor and the outcome [46,47]. In the context of the current study, it is possible that income is negatively influenced by autozygosity and positively influenced by intelligence. If so, then the regression coefficient for *F*_*ROH*_ predicting fluid intelligence would be upwardly biased when income is included as a covariate. Unfortunately, it was not possible to know the degree to which our results were influenced by a collider bias given that the directions of causation between variables are unknown. Nevertheless, it has been argued that the potential bias from failing to adjust for a covariate is likely to be greater than the bias introduced when controlling for a collider [48,49]. Thus, our solution was to present results from models that both controlled and did not control for the sociodemographic covariates, and to highlight the potential for biases in models that controlled for sociodemographic variables.

The third limitation to the current study is that we did not have access to all of the phenotypes studied in recent articles such as Yengo et al. [10], Verweij et al. [9], Joshi et al. [7] (e.g. the cholesterol measures in Joshi et al.), so we could not attempt to fully replicate these previous investigations. In addition, as mentioned earlier, the covariate that we had available as a proxy for religiosity was unlikely to account for all possible confounding of religiosity on *F*_*ROH*_-trait associations.

### Summary

We found several significant associations between estimated autozygosity and several sociodemographic, anthropometric, health, and otherwise fitness-related traits. All effects were in the direction that would be predicted by evolutionary hypotheses (i.e. higher inbreeding associated with lower fitness). When controlling for measures of background sociodemographic characteristics (educational attainment, college education, income, urbanicity, TDI, religious participation, and whether an individual was breastfed)–which should at least partially reflect parental characteristics–we found that two (height and grip strength) of the five significant *F*_*ROH*_-trait associations were attenuated and became non-significant, while AFS, FI, and FEV1 remained significantly associated with *F*_*ROH*_. The fact that the associations between estimated autozygosity and both grip strength and height were reduced after controlling for the additional covariates suggests that these relationships might not hold up if relevant confounder variables in parents had been controlled for, and we cannot eliminate the possibility that the other *F*_*ROH*_-trait associations we report here would not also be attenuated or eliminated in this situation.

Nevertheless, our results generally replicate previous findings in humans [7–9] and are consistent with similar ones from non-human populations [40,50,51]. This cumulative evidence may well reflect the detrimental effects of autozygosity on complex traits, revealing ancient selection pressures on these or correlated traits. However, the fact remains that even in very large, well-powered, unascertained samples such as this one, it is exceedingly difficult to make definitive statements about the underlying causal mechanism of observed relationships between *F*_*ROH*_ and complex traits.

## Supporting information

S1 Text Additional information on phenotype derivation, choice of autozygosity detection method and parameters, *F*_*ROH_long*_ vs. *F*_*ROH_short*_, mediation analysis and testing for indirect effect, *F*_*SNP*_ simulations. (DOCX)

S1 Table Descriptive statistics of the UK Biobank sample–continuous variables. (DOCX)

S2 Table Descriptive statistics of the UK Biobank sample–binary variables. (DOCX)

S3 Table Correlations between *F*_*ROH*_ from longer ROHs (*F*_*ROH_long*_, indicative of more recent inbreeding), *F*_*ROH*_ from shorter ROHs (*F*_*ROH_short*_, a proxy for more distant inbreeding), and *F*_*SNP*_, a measure of excess homozygosity at common SNPs. (DOCX)

S4 Table Results from simulations of fully recessive quantitative phenotypes caused exclusively by homozygosity at either common (minor allele frequency (MAF) > 0.05) or rare (MAF < 0.05) variants. Linear regression models included both *F*_*ROH*_ and *F*_*SNP*_, as well as age, age^2^, sex, batch number, sample missingness, and the first 20 principle components. We report the average Beta and p-value across the 100 simulations for each MAF class of causal variants (CVs).
(DOCX)

S5 Table **Association of *F***_***ROH***_ **with 26 traits in smaller sample of individuals unrelated at pihat > 0.05, for two sets of models: 1) controlling for age, age**^**2**^**, sex, the first 20 principal components, sample missingness, and batch number as covariates, and 2) also controlling for sociodemographic variables.** The quantitative traits (analyzed via linear regression) are listed first in the table, followed by diagnoses and binary traits (analyzed via logistic regression models). Phenotypes with a significant relationship with *F*_*ROH*_ (*p* < 0.002 after multiple testing correction) are bolded; those with an asterisk are also significantly associated with *F*_*ROH*_ after controlling for sociodemographic covariates (income, educational attainment, college degree, urbanicity, TDI, religious group participation, and whether or not they were breastfed as an infant). BP, blood pressure; FEV1, forced expiratory volume in 1 second; FVC, forced vital capacity; BPD, bipolar disorder; MDD, major depressive disorder; df, degrees of freedom; SE, standard error.
(DOCX)

S6 Table Effects of *F*_*ROH*_ from recent and distant inbreeding (analyzed in two separate models), defined as the proportion of the genome contained in autozygous segments longer than or shorter than 8.5 Mb, respectively. All models controlled for age, age^2^, sex, batch number, per-sample SNP missingness, and the first 20 principal components. Phenotypes with a significant relationship (*p* < 0.002 after multiple testing correction) with *F*_*ROH*_ from recent inbreeding are bolded, while those with a significant relationship with *F*_*ROH*_ from distant inbreeding are starred. The quantitative traits (analyzed via linear regression) are listed first in the table, followed by diagnoses and binary traits (analyzed via logistic regression models). BP, blood pressure; FEV1, forced expiratory volume in 1 second; FVC, forced vital capacity; BPD, bipolar disorder; MDD, major depressive disorder; df, degrees of freedom; SE, standard error.
(DOCX)

S7 Table Effects of F_ROH_ from recent and distant inbreeding, when both F_ROH_long_ and F_ROH_short_ are simultaneously entered into the regression model. All models controlled for age, age2, sex, batch number, per-sample SNP missingness, and the first 20 principal components. Phenotypes with a significant association (p < 0.002 after multiple testing correction) with FROH_long are bolded, while those with a significant relationship with FROH_short are starred. The quantitative traits (analyzed via linear regression) are listed first in the table, followed by diagnoses and binary traits (analyzed via logistic regression models). BP, blood pressure; FEV1, forced expiratory volume in 1 second; FVC, forced vital capacity; BPD, bipolar disorder; MDD, major depressive disorder; df, degrees of freedom; SE, standard error.
(DOCX)

S8 Table Effects of *F*_*ROH*_ from recent and distant inbreeding (analyzed in two separate models), controlling for background sociodemographic characteristics. All models controlled for age, age^2^, sex, batch number, per-sample SNP missingness, the first 20 principal components, and seven sociodemographic variables. Phenotypes with a significant relationship (*p* < 0.003 after multiple testing correction) with *F*_*ROH*_ from recent inbreeding are bolded, while those with a significant relationship with *F*_*ROH*_ from distant inbreeding are starred. The quantitative traits (analyzed via linear regression) are listed first in the table, followed by diagnoses and binary traits (analyzed via logistic regression models). BP, blood pressure; FEV1, forced expiratory volume in 1 second; FVC, forced vital capacity; BPD, bipolar disorder; MDD, major depressive disorder; df, degrees of freedom; SE, standard error.
(DOCX)

S9 Table Effects of both *F*_*ROH*_ and excess SNP-by-SNP homozygosity, measured by *F*_*SNP*_, in smaller sample of individuals unrelated at pihat > 0.05, controlling for the covariates in the previous models (age, age^2^, sex, batch number, per-sample SNP missingness, the first 20 principal components, and background sociodemographic variables.). Phenotypes with a significant relationship with *F*_*ROH*_ (*p* < 0.003 after multiple testing correction) are bolded, while those with a significant relationship with *F*_*SNP*_ are italicized. The quantitative traits (analyzed via linear regression) are listed first in the table, followed by diagnoses and binary traits (analyzed via logistic regression models). BP, blood pressure; FEV1, forced expiratory volume in 1 second; FVC, forced vital capacity; BPD, bipolar disorder; MDD, major depressive disorder; df, degrees of freedom; SE, standard error.
(DOCX)

S1 Fig Distribution of ROH lengths in Kb (left) and *F*_*ROH*_ (right) in the UK Biobank sample. The histogram of ROH lengths is cut off at 6500 Kb for clarity; similarly, the histogram of *F*_*ROH*_ is cut off at 0.05 for clarity. There were 583 individuals who had *F*_*ROH*_ > 0.05; these individuals were not excluded from analyses.
(DOCX)

S2 Fig Distribution of *F*_*SNP*_ in the UK Biobank sample. The histogram of FSNP is cut off at |0.01| for clarity; 77 individuals had levels of SNP-by-SNP homozygosity greater than 0.1 or less than -0.1.
(DOCX)
