# Supplementary material for: Relationships between estimated autozygosity and complex traits in the UK Biobank
Source: PLoS Genet. 2018 Jul 27;14(7):e1007556. doi: 10.1371/journal.pgen.1007556 (PMC6082573; doi:10.1371/journal.pgen.1007556)
Supplement: S1 Text — (DOCX) [file pgen.1007556.s001.docx]

**Supplemental Materials**

**UK Biobank Phenotype Details**

We examined 26 traits from the UK Biobank broadly related to health, fitness, or sociodemographic characteristics:

*Age at first sexual intercourse*: Field 2139 in the UK Biobank (sensitive information). Answers to the touchscreen interview question “What was your age when you first had sexual intercourse? (Sexual intercourse includes vaginal, oral or anal intercourse)". Units of measurement are years. Individuals who reported having had sex before 12 years of age were excluded from our analyses.

*Basal metabolic rate (BMR)*: Field 23105, body composition estimated with impedance measurement; units are KJ.

*Birth weight*: Field 20022, from the verbal interview. Participants were asked to give their birth weight, with measurement units in kg. For the current analyses, we excluded individuals who reported a birth weight greater than 6.8 kg (~15 lbs), in an effort to exclude incorrect entries or unique situations that might reflect something beyond typical human variation.

*Probable bipolar episodes (BPD)*: This measure was derived from Field 20126, which was in turn derived from Fields 20122 – 20125 by Prof. Jill Pell (more information at http://biobank.ctsu.ox.ac.uk/crystal/refer.cgi?id=158772). Briefly, individuals who completed all the touchscreen questions regarding these mental health symptoms were categorized into one of six categories: 0 = no bipolar or depression; 1 = Bipolar I; 2 = Bipolar II; 3 = Probable Recurrent major depression (severe); 4 = Probable Recurrent major depression (moderate); 5 = Single Probable major depression episode. If an individual’s responses resulted in their being coded as possessing more than one disorder, they were only categorized as their most severe condition (e.g. if an individual qualified for Bipolar II as well as probable severe recurrent major depression, they were coded as 2, Bipolar II, for derived Field 20126). For our analyses, any individual who was coded as either 1 or 2 (“Bipolar I” or “Bipolar II”) for Field 20126 was counted as a case (coded as ‘1’), and any individual coded as 0 (“No bipolar or depression”) was included as a control (‘0’).

*Body mass index (BMI)*: Field 21001, units in kg/m^2^.

*Body fat percentage*: Field 23099.

*Diabetes diagnosed by a doctor*: Field 2443. Individuals were given the options of “Yes”, “No”, “Prefer not to answer”, and “Do not know”. For the current study, individuals who responded “Yes” were included as cases (coded as ‘1’), and those who answered “No” were considered controls (‘0’).

*Breastfed as a baby*: Field 1677 from the touchscreen interview, “Were you breastfed when you were a baby?” Possible responses were “Yes”, “No”, “Do not know”, “Prefer not to answer”. For the current study, we only included individuals who answered either “Yes” (coded as ‘1’), or “No” (coded as ‘0’).

*Completed college/university*: This measure was derived from years of educational attainment, which is discussed in more detail below. Briefly, individuals who reported having a college or university degree were coded as ‘1’, and those who did not were coded as ‘0’.

*Diastolic blood pressure (BP)*: From Field 4079, which included two automated diastolic blood pressure readings, taken a few moments apart. For this analysis, we took the average of the two readings (or just included one reading, if only one was given) for each individual, and excluded those with an average diastolic BP reading > 120 mmHg (the American Heart Association classifies a diastolic BP reading > 110 mmHg as a hypertensive crisis, requiring immediate emergency care).

*Educational attainment*: Derived from Field 6138, “Qualifications”, during the touchscreen interview. Individuals were asked “Which of the following qualifications do you have? (You can select more than one)”, and could choose from among the following possible responses: College or University degree; A levels/AS levels or equivalent; O levels/GCSEs or equivalent; CSEs or equivalent; NVQ or HND or HNC or equivalent; Other professional qualifications eg: nursing, teaching; None of the above; Prefer not to answer. For the current study, we extracted each individual’s highest level of education completed, converted those qualifications to International Standard Classification of Education (ISCED) codes, and then translated each individual’s ISCED code into years of educational attainment as in Supplementary Table 1.14 of Okbay et al. (2016) ^1^.

*Ever drink*: This measure came from Field 20117, “Alcohol Drinker Status”, from the touchscreen interview. Calculated based on participants’ answers to questions like “About how often do you drink alcohol?” and “Did you previously drink alcohol?”, individuals were categorized into “Current”, “Previous”, “Never”, and “Prefer not to answer”. For the current analyses, we classified individuals in the “Current” or “Previous” categories as “ever drinkers” (coded as ‘1’), and individuals in the “Never” category as “never drinkers” (‘0’).

*Ever smoke*: This measure came from Field 20116, “Smoking Status”, which was derived by the UK Biobank from Fields 1239 (“Current tobacco smoking”) and 1249 (“Past tobacco smoking”). Based on their responses to those two fields, individuals were categorized into “Current”, “Previous”, “Never”, and “Prefer not to answer”. For the current analyses, we classified individuals in the “Current” or “Previous” categories as “ever smokers” (coded as ‘1’), and individuals in the “Never” category as “never smokers” (‘0’).

*Fluid intelligence*: Field 20016, from the cognitive function assessment. This measure is the unweighted sum of the number of 13 fluid intelligence questions answered correctly.

*Forced expiratory volume in 1 second (FEV1) and FEV1/forced vital capacity (FVC)*: FEV1 came from Field 20150, which was the “Highest measure from the array of values for Forced Expiratory Volume in 1-second (FEV1) Field 3063, which was "acceptable" according to corresponding acceptability field (Field 3061).” This field was derived by Prof. Martin Tobin, and the units of measurement are liters. FVC came from Field 20151, which was also derived by Prof. Tobin in a similar manner. For the current study, our measure of FEV1/FVC was simply calculated as the ratio of these two fields.

*Grip strength*: This measure was derived from Fields 46, left hand grip strength, and 47, right hand grip strength; units of measurement are kg. We calculated maximum grip strength for each individual by taking the maximum value from either left or right hand grip strength, as was done in reference ^2^. We excluded individuals with hand grip strength values >70 kg, as these were obvious outliers.

*Height*: This measurement came from Field 50, “Standing height”, and units of measurement are cm. We excluded individuals who stood less than 120 cm high.

*Household income*: Field 738, from the touchscreen question “What is the average total income before tax received by your HOUSEHOLD?" Possible response categories were: Less than £18,000; £18,000 to £30,999; £31,000 to £51,999; £60,000 to £100,000; greater than £100,000; Do not know; Prefer not to answer. We excluded individuals who responded “Do not know” or “Prefer not to answer”, and treated the remaining categories continuously, such that “Less than £18,000” was coded as 0, “£18,000 to £30,999” was coded as 1, etc.

*Probable major depression (MDD)*: This measure was derived from Field 20126, which was in turn derived from Fields 20122 – 20125 by Prof. Jill Pell (more information at http://biobank.ctsu.ox.ac.uk/crystal/refer.cgi?id=158772). Briefly, individuals who completed all the touchscreen questions regarding these mental health symptoms were categorized into one of six categories: 0 = no bipolar or depression; 1 = Bipolar I; 2 = Bipolar II; 3 = Probable Recurrent major depression (severe); 4 = Probable Recurrent major depression (moderate); 5 = Single Probable major depression episode. If an individual’s responses resulted in their being coded as possessing more than one disorder, they were only categorized as their most severe condition (e.g. if an individual qualified for Bipolar II as well as probable severe recurrent major depression, they were coded as 2, Bipolar II, for derived Field 20126). For our analyses, any individual who was coded as 3, 4, or 5 (“Probable Recurrent major depression (severe)”, ”Probable Recurrent major depression (moderate)”, or “Single Probable major depression episode”) for Field 20126 was counted as a case (coded as ‘1’), and any individual coded as 0 (“No bipolar or depression”) was included as a control (‘0’).

*Neuroticism score:* This score, derived by Prof. Jill Pell, was calculated from 12 “neurotic behavior domains” from fields 1920, 1930, 1940, 1950, 1960, 1970, 1980, 1990, 2000, 2010, 2020 and 2030; possible scores range from 0 to 12. More details are provided at <http://biobank.ctsu.ox.ac.uk/crystal/field.cgi?id=20127>.

*Religious group attendance/participation:* We derived this score from Field 6160, a touchscreen question that asked "Which of the following do you attend once a week or more often? (You can select more than one)". Any individual who selected “Religious group” as one of their answers was coded as a ‘1’ for our measure, while everyone else who did not select this as an answer was coded as a ‘0’.

*Systolic blood pressure (BP):* From Field 4080, which included two automated systolic blood pressure readings, taken a few moments apart. For this analysis, we took the average of the two readings (or just included one reading, if only one was given) for each individual, and excluded those with an average systolic BP reading > 200 mmHg (the American Heart Association classifies a systolic BP reading > 180 mmHg as a hypertensive crisis, requiring immediate emergency care).

*Townsend Deprivation Index (TDI):* Field 189, calculated by the UK Biobank based on an individual’s postcode and the TDI score assigned to output areas by the preceding national census.

*Urbanicity:* This binary measure was derived from Field 20118, “Home area population density – urban or rural”, which was a classification originally created by combining participants’ home postal code with population density data from the 2001 census (from the Office of National Statistics). We converted this field into a dichotomous measure by combining the four urban categories (“England/Wales – Urban – sparse”, “England/Wales – Urban – less sparse”, “Scotland – Large Urban Area”, and “Scotland – Other Urban area”) into one urban category (coded ‘1’), and all other classifications (e.g. “England/Wales – Town and Fringe – sparse”, “Scotland – Remote Small Town”) into a non-urban category (coded ‘0’).

*Waist:hip ratio:* This was calculated from Fields 48 and 49, waist and hip circumference in cm. We excluded individuals with a waist circumference <40 cm or >160 cm or a hip circumference <50 cm or >175 cm (i.e. individuals who were extreme outliers), and then took the ratio of waist circumference to hip circumference.

**Choice of autozygosity detection method**

As Yengo et al.^3^ discuss in their paper comparing methods for the quantification of inbreeding in the UK Biobank, one shortcoming of ROH-based methods is that the ROH definition depends on the relatively arbitrary choice of multiple parameters, including whether to define ROH based on physical length or number of consecutive homozygous SNPs, the number of missing or heterozygote SNPs allowed per ROH, and the maximum gap allowed between two SNPs in the ROH. Despite these many parameter choices, *F_ROH_* has been shown to be the best estimate of genome-wide autozygosity^4,5^ (comparable to measurements from pedigree data), and Howrigan et al.^6^ clearly defined the optimum parameters for autozygosity detection using the suite of --homozyg commands implemented in Plink^7^, basing their recommendations on extensive simulations of realistic SNP data from European-ancestry populations. In particular, Howrigan et al. recommended pruning SNPs in strong linkage disequilibrium (LD) before calling ROHs, as regions of high LD can result in an increase of false positive ROH calls. Howrigan et al. then specified a set of optimum ROH-calling parameters for data with light LD pruning (removal of SNPs with VIF $\geq$ 10 within a 50-SNP window) or more moderate LD pruning (removal of SNPs within a 50-SNP window with VIF $\geq$ 2). We followed their recommendations for light LD pruning, specifically requiring ROHs to consist of at least 65 consecutive homozygous SNPs in a row, have a density of at least 1 SNP per 200 kb, no heterozygote calls and three missing variant calls (5% of the SNP threshold) per ROH. Finally, we split an ROH into two if a gap >500 kb existed between consecutive homozygous SNPs. In contrast, Joshi et al. defined ROHs on a set of SNPs that were not LD-pruned, but required ROHs to be at least 1.5 Mb long, to contain at least 50 consecutive homozygous SNPs, and to be located at least 1 Mb away from one another. Joshi et al.’s shorter ROH SNP threshold might capture some ancient segments of autozygosity that will be missed by our choice of ROH definition, but this definition is also more prone to calling false positives (regions that are homozygous by state, rather than identical by descent), particularly on unpruned SNP data.

***F_ROH_long_* vs. *F_ROH_short_***

As the effect of *F_ROH_short_* (distant inbreeding) controlling for *F_ROH_long_* (recent inbreeding) might be seen as a way to estimate the effects of autozygosity while controlling for assortative mating (i.e. recent inbreeding) without the need to include sociodemographic covariates, we conducted post-hoc analyses where both *F_ROH_short_* and *F_ROH_long_* were entered into the regression model as predictors simultaneously. These results are presented in Table S7; only AFS and religious group participation were significantly associated with *F_ROH_short_* in this model. Unfortunately, this method has two major drawbacks: first, because of the high collinearity between *F_ROH_short_* and *F_ROH_long_*, the standard errors of these predictors in the model are larger than when *F_ROH_short_* and *F_ROH_long_* are tested in separate models. Second, although there is an average difference in time to most recent common ancestor when comparing *F_ROH_short_* to *F_ROH_long_*, recent inbreeding can also produce many short ROHs (as ROHs are exponentially distributed). Thus, while calculating *F_ROH_* from short ROHs is likely a better estimate of true autozygosity due to distant inbreeding, it is not a perfectly accurate method, nor is it exempt from potential confounding due to recent inbreeding caused by e.g. assortative mating.

**Testing for significant indirect mediation effect of the sociodemographic variables on *F_ROH_* – trait relationships**

To formally test whether the seven sociodemographic variables, in combination, significantly mediated the *F_ROH_* associations identified in the first model, we followed Kenny and Judd’s recommendations^8^ for calculating the indirect effect size and then bootstrapped with 1,000 resamples to get the 99% confidence intervals around the indirect path coefficients for any significant *F_ROH_* – trait associations we observed in our first set of models. Specifically, Judd and Kenny recommend calculating the indirect effect by a difference of coefficients method. If Model 1 is $Y= \beta_{0}+ \beta_{1}X+ \beta_{2}M+e$, and Model 2 is given as $Y=\beta_{0}+ \beta X+e$ , then the indirect effect of the mediator *M* on the relationship between *X* and *Y* can be calculated as $\beta_{indirect}= \beta- \beta_{1}$. In the current study, we included the 7 sociodemographic variables in the regression model as ‘M’, so our $\beta_{indirect}$ was calculated by subtracting $\beta_{FROH}$in the “full” model (with sociodemographic covariates) from $\beta_{FROH}$in the “simple” model (no sociodemographic covariates). We then tested the significance of $\beta_{indirect}$ by using percentile bootstrapping to obtain the standard error and 95% confidence intervals around the $\beta_{indirect}$ estimate (analyses conducted using the ‘boot’ package in R). We found no significant indirect effects for any of the five traits, suggesting that the relationship between *F_ROH_* and these traits is only weakly mediated by subjects’ sociodemographic characteristics.

**Simulations testing whether *F_SNP_* and *F_ROH_* differentially associate with complex traits caused by homozygosity at rare vs. common variants**

### To ensure that our regression models including both *F_ROH_* and *F_SNP_* as predictors were capturing the effects of homozygosity at common versus rare loci as we expected, we simulated a fully recessive quantitative trait from 500 causal variants (CVs), randomly chosen from either a) common genotyped SNPs (MAF > 0.05), or b) less common genotyped SNPs (MAF < 0.05). We then called ROHs from the set of non-CV SNPs (after performing typical QC procedures and LD pruning, as described in the Methods) and regressed the simulated phenotype on *F_ROH_*, *F_SNP_*, and the typical covariates (age, age^2^, sex, the 1^st^ 20 PCs, SNP missingness, and genotype batch). We repeated this process 100 times, and report the average β and *p-*value for both *F_ROH_* and *F_SNP_* in each model in Table S4. Our results showed that for the simulated trait caused only by homozygosity at common variants, *F_SNP_* was more significantly associated with the trait than *F_ROH_*, though both predictors were significant in the regression model. For a trait determined by homozygosity at rare variants, *F_ROH_* was a much more significant predictor of trait variance than *F_SNP_*. This confirmed our expectations that, in a model controlling for *F_SNP,_* if a trait is caused largely by homozygosity at rare, recessive variants (as expected under a model of purifying selection), *F_ROH_* will capture the trait variance caused by homozygosity at these rare CVs that *F_SNP_* is unable to measure.

1. Okbay, A. *et al.* Genome-wide association study identifies 74 loci associated with educational attainment. *Nature* **533,** 539–542 (2016).

2. Willems, S. M. *et al.* Large-scale GWAS identifies multiple loci for hand grip strength providing biological insights into muscular fitness. *Nat. Commun.* **8,** 16015 (2017).

3. Yengo, L. *et al.* Detection and quantification of inbreeding depression for complex traits from SNP data. *Proc. Natl. Acad. Sci.* **114,** 8602 LP-8607 (2017).

4. Keller, M. C., Visscher, P. M. & Goddard, M. E. Quantification of inbreeding due to distant ancestors and its detection using dense single nucleotide polymorphism data. *Genetics* **189,** 237–249 (2011).

5. Gazal, S. *et al.* Inbreeding coefficient estimation with dense SNP data: Comparison of strategies and application to HapMap III. *Hum. Hered.* **77,** 49–62 (2014).

6. Howrigan, D. P., Simonson, M. A. & Keller, M. C. Detecting autozygosity through runs of homozygosity: A comparison of three autozygosity detection algorithms. *BMC Genomics* **12,** 460 (2011).

7. Purcell, S. *et al.* PLINK: a tool set for whole-genome association and population-based linkage analyses. *Am. J. Hum. Genet.* **81,** 559–575 (2007).

8. Judd, C. M. & Kenny, D. A. Process analysis: Estimating mediation in treatment evaluations. *Eval. Rev.* **5,** 602–619 (1981).
