# Supplementary material for: Relationships between estimated autozygosity and complex traits in the UK Biobank
Source: PLoS Genet. 2018 Jul 27;14(7):e1007556. doi: 10.1371/journal.pgen.1007556 (PMC6082573; doi:10.1371/journal.pgen.1007556)
Supplement: S1 Table — (DOCX) [file pgen.1007556.s002.docx]

|  | **N** | **Units** | **Mean** | **SD** | **Min** | **Max** |
| --- | --- | --- | --- | --- | --- | --- |
| **F_ROH_** | 404518 | Sum(ROHs)/total SNP-mappable genome length | 0.004 | 0.004 | 0 | 0.211 |
| **Age** | 404518 | Years | 65.837 | 7.958 | 47 | 83 |
| **Income** | 347883 | Categorical - divided into 5 brackets (0 - 4) based on household yearly income before tax | 1.614 | 1.188 | 0 | 4 |
| **Educational attainment** | 400383 | Years of education according to ISCED codes | 14.868 | 5.114 | 7 | 20 |
| **TDI** | 404034 | Townsend Deprivation Index score | 6.250 | 2.968 | -6.258 | 11.001 |
| **AFS** | 354311 | Years | 19.125 | 3.806 | 12 | 69 |
| **BMR** | 397363 | KJ | 6644.661 | 1369.804 | 3531 | 15506 |
| **Birth weight** | 229569 | Kg | 3.319 | 0.667 | 0.450 | 6.8 |
| **BMI** | 403173 | Kg/m2 | 59.388 | 4.764 | 12.121 | 74.684 |
| **Body fat percentage** | 397148 | Percent body fat | 43.416 | 8.513 | 5.0 | 69.8 |
| **Diastolic BP** | 380686 | mmHg | 82.233 | 10.070 | 32 | 120 |
| **Systolic BP** | 379733 | mmHg | 137.983 | 18.217 | 72 | 200 |
| **FEV1** | 304301 | Litres | 2.856 | 0.780 | 0.090 | 14.650 |
| **FEV1/FVC** | 304301 | Litres | 0.754 | 0.066 | 0.082 | 2.177 |
| **Height** | 403609 | cm | 168.792 | 9.237 | 121 | 209 |
| **Grip strength** | 403589 | Kg | 32.833 | 11.281 | 0 | 70 |
| **Waist-to-hip ratio** | 403689 | cm | 0.872 | 0.090 | 0.448 | 1.559 |
| **FI** | 145658 | Number correct answers out of 13 | 6.234 | 2.119 | 0 | 13 |
| **Neuroticism score** | 327994 | Sum score of 12 questions | 4.109 | 3.259 | 0 | 12 |
