# Supplementary material for: Relationships between estimated autozygosity and complex traits in the UK Biobank
Source: PLoS Genet. 2018 Jul 27;14(7):e1007556. doi: 10.1371/journal.pgen.1007556 (PMC6082573; doi:10.1371/journal.pgen.1007556)
Supplement: S2 Table — (DOCX) [file pgen.1007556.s003.docx]

|  | **N** | **N case/yes** | **N control/no** |
| --- | --- | --- | --- |
| **College completion** | 404518 | 127985 | 276533 |
| **Lives in urban area** | 400629 | 340652 | 59977 |
| **Religious group participation** | 404518 | 59111 | 345407 |
| **Breastfed as infant** | 305904 | 217036 | 88868 |
| **Diabetes diagnosis** | 403387 | 19555 | 383832 |
| **Ever drink** | 403990 | 391380 | 12610 |
| **Ever smoke** | 365395 | 219865 | 145530 |
| **Probable BPD** | 71007 | 1177 | 69830 |
| **Probable MDD** | 95481 | 25651 | 69830 |
