# Supplementary material for: Relationships between estimated autozygosity and complex traits in the UK Biobank
Source: PLoS Genet. 2018 Jul 27;14(7):e1007556. doi: 10.1371/journal.pgen.1007556 (PMC6082573; doi:10.1371/journal.pgen.1007556)
Supplement: S3 Table — (DOCX) [file pgen.1007556.s004.docx]

|  | **F_ROH_short_** | **F_ROH_long_** |
| --- | --- | --- |
| **F_ROH_long_** | 0.469 | - |
| **F_SNP_** | 0.483 | 0.480 |
