# Supplementary material for: Relationships between estimated autozygosity and complex traits in the UK Biobank
Source: PLoS Genet. 2018 Jul 27;14(7):e1007556. doi: 10.1371/journal.pgen.1007556 (PMC6082573; doi:10.1371/journal.pgen.1007556)
Supplement: S4 Table — Linear regression models included both FROH and FSNP, as well as age, age2, sex, batch number, sample missingness, and the first 20 principle components. We report the average Beta and p-value across the 100 simulations for each MAF class of causal variants (CVs). (DOCX) [file pgen.1007556.s005.docx]

| **MAF of CVs** | **Beta_Froh_** | **p_Froh_** | **Beta_Fsnp_** | **p_Fsnp_** |
| --- | --- | --- | --- | --- |
| > 0.05 | 8.34 | 3.85E-39 | 6.83 | 1.60E-90 |
| < 0.05 | 13.55 | 8.63E-109 | 1.70 | 1.36E-05 |
