# Supplementary material for: Relationships between estimated autozygosity and complex traits in the UK Biobank
Source: PLoS Genet. 2018 Jul 27;14(7):e1007556. doi: 10.1371/journal.pgen.1007556 (PMC6082573; doi:10.1371/journal.pgen.1007556)
Supplement: S5 Table — Association of FROH with 26 traits in smaller sample of individuals unrelated at pihat > 0.05, for two sets of models: 1) controlling for age, age2, sex, the first 20 principal components, sample missingness, and batch number as covariates, and 2) also controlling for sociodemographic variables. The quantitative traits (analyzed via linear regression) are listed first in the table, followed by diagnoses and binary traits (analyzed via logistic regression models). Phenotypes with a significant relationship with FROH (p < 0.002 after multiple testing correction) are bolded; those with an asterisk are also significantly associated with FROH after controlling for sociodemographic covariates (income, educational attainment, college degree, urbanicity, TDI, religious group participation, and whether or not they were breastfed as an infant). BP, blood pressure; FEV1, forced expiratory volume in 1 second; FVC, forced vital capacity; BPD, bipolar disorder; MDD, major depressive disorder; df, degrees of freedom; SE, standard error. (DOCX) [file pgen.1007556.s006.docx]

|  |  |  | **Main models - controlling for batch, sample missingness, sex, age, age^2^, and first 20 principle components** | | | **Models also controlling for sociodemographic covariates (income, educational attainment, college, urban, TDI, religiosity, breastfed)** | | |
| --- | --- | --- | --- | --- | --- | --- | --- | --- |
| **Category** | **Trait** | **df** | **Beta** | **SE** | **p** | **Beta** | **SE** | **p** |
| **Quantitative Traits (linear regression)** | | | | | | | | |
| Sociodemographic | **income** | 289767 | -1.835 | 0.524 | 4.66E-04 |  |  |  |
| Sociodemographic | years of education | 332807 | -0.321 | 0.504 | 0.524 |  |  |  |
| Sociodemographic | Townsend Deprivation Index | 335759 | -0.913 | 0.508 | 0.072 |  |  |  |
| biometric | basal metabolic rate | 330293 | -0.929 | 0.510 | 0.068 | -0.591 | 0.672 | 0.379 |
| biometric | birth weight | 191544 | -0.950 | 0.698 | 0.173 | -1.197 | 0.795 | 0.132 |
| biometric | body mass index | 335040 | -0.537 | 0.513 | 0.295 | -0.472 | 0.664 | 0.477 |
| biometric | body fat percentage | 330106 | -0.462 | 0.511 | 0.367 | -0.185 | 0.665 | 0.780 |
| biometric | diastolic BP | 316483 | 0.879 | 0.531 | 0.098 | 1.358 | 0.692 | 0.050 |
| biometric | systolic BP | 315721 | 0.843 | 0.507 | 0.096 | 0.479 | 0.652 | 0.462 |
| biometric | **forced expiratory volume in 1 second (FEV1)*** | 253192 | -2.647 | 0.538 | 8.61E-07 | -2.446 | 0.689 | 3.89E-04 |
| biometric | FEV1/FVC | 253192 | -0.775 | 0.596 | 0.193 | 0.296 | 0.757 | 0.696 |
| biometric | **height** | 335406 | -1.917 | 0.501 | 1.29E-04 | -1.277 | 0.648 | 0.049 |
| biometric | **grip strength** | 335404 | -1.851 | 0.493 | 1.72E-04 | -1.266 | 0.643 | 0.049 |
| biometric | waist to hip ratio | 335468 | -1.398 | 0.505 | 0.006 | -1.501 | 0.652 | 0.021 |
| health- and fitness-related | **age at first sexual intercourse*** | 294916 | 4.313 | 0.558 | 1.06E-14 | 3.071 | 0.673 | 5.08E-06 |
| health- and fitness-related | **fluid intelligence*** | 122547 | -3.448 | 0.832 | 3.43E-05 | -3.388 | 0.969 | 4.72E-04 |
| health- and fitness-related | neuroticism score | 272861 | -0.449 | 0.570 | 0.431 | -0.171 | 0.723 | 0.813 |
| **Binary Outcomes (logistic regression)** | | | | | | | | |
| Sociodemographic | breastfed as infant | 255113 | -1.852 | 1.389 | 0.182 |  |  |  |
| Sociodemographic | college degree | 336165 | 0.325 | 1.113 | 0.770 |  |  |  |
| Sociodemographic | live in urban area | 332948 | -1.391 | 1.396 | 0.319 |  |  |  |
| Sociodemographic | **religious group attendance** | 336165 | 8.549 | 1.233 | 4.08E-12 |  |  |  |
| health- and fitness-related | diagnosed with diabetes | 335230 | -0.345 | 2.339 | 0.883 | 0.138 | 3.155 | 0.965 |
| health- and fitness-related | ever drink | 335715 | 4.964 | 2.433 | 0.041 | 1.060 | 3.693 | 0.774 |
| health- and fitness-related | ever smoke | 303916 | 1.985 | 1.136 | 0.081 | 1.463 | 1.498 | 0.329 |
| health- and fitness-related | Probable BPD diagnosis | 59626 | 6.551 | 11.572 | 0.571 | 12.796 | 16.515 | 0.438 |
| health- and fitness-related | Probable MDD diagnosis | 80257 | 0.240 | 2.309 | 0.917 | 0.140 | 2.851 | 0.961 |
