# Supplementary material for: Relationships between estimated autozygosity and complex traits in the UK Biobank
Source: PLoS Genet. 2018 Jul 27;14(7):e1007556. doi: 10.1371/journal.pgen.1007556 (PMC6082573; doi:10.1371/journal.pgen.1007556)
Supplement: S6 Table — All models controlled for age, age2, sex, batch number, per-sample SNP missingness, and the first 20 principal components. Phenotypes with a significant relationship (p < 0.002 after multiple testing correction) with FROH from recent inbreeding are bolded, while those with a significant relationship with FROH from distant inbreeding are starred. The quantitative traits (analyzed via linear regression) are listed first in the table, followed by diagnoses and binary traits (analyzed via logistic regression models). BP, blood pressure; FEV1, forced expiratory volume in 1 second; FVC, forced vital capacity; BPD, bipolar disorder; MDD, major depressive disorder; df, degrees of freedom; SE, standard error. (DOCX) [file pgen.1007556.s007.docx]

|  |  |  | **Recent Inbreeding Models (*Froh* from ROHs > 8.5 Mb)** | | | **Distant Inbreeding Models (*Froh* from ROHs < 8.5 Mb)** | | |
| --- | --- | --- | --- | --- | --- | --- | --- | --- |
| **Category** | **Trait** | **df** | **Beta** | **SE** | **p** | **Beta** | **SE** | **p** |
| **Quantitative Traits (linear regression)** | | | | | | | | |
| Sociodemographic | **income** | 347883 | -4.183 | 0.760 | 3.74E-08 | -0.622 | 0.768 | 0.418 |
| Sociodemographic | years of education | 400383 | -0.748 | 0.725 | 0.302 | -0.447 | 0.747 | 0.550 |
| Sociodemographic | Townsend Deprivation Index | 404034 | -1.883 | 0.732 | 0.010 | 0.217 | 0.757 | 0.774 |
| biometric | basal metabolic rate | 397363 | -1.047 | 0.734 | 0.154 | -1.686 | 0.757 | 0.026 |
| biometric | birth weight | 229569 | -2.784 | 0.994 | 0.005 | 0.158 | 1.024 | 0.878 |
| biometric | body mass index | 403173 | -1.474 | 0.738 | 0.046 | 0.148 | 0.764 | 0.846 |
| biometric | body fat percentage | 397148 | -1.589 | 0.736 | 0.031 | 0.053 | 0.759 | 0.945 |
| biometric | diastolic BP | 380686 | 1.998 | 0.762 | 0.009 | 0.486 | 0.789 | 0.538 |
| biometric | systolic BP | 379733 | 1.873 | 0.728 | 0.010 | 0.316 | 0.752 | 0.674 |
| biometric | **forced expiratory volume in 1 second (FEV1)*** | 304301 | -4.280 | 0.787 | 5.35E-08 | -3.577 | 0.783 | 4.98E-06 |
| biometric | FEV1/FVC | 304301 | -1.517 | 0.871 | 0.082 | -0.203 | 0.868 | 0.815 |
| biometric | **height** | 403609 | -3.273 | 0.720 | 5.53E-06 | -2.046 | 0.745 | 0.006 |
| biometric | **grip strength** | 403589 | -2.800 | 0.708 | 7.66E-05 | -2.190 | 0.731 | 0.003 |
| biometric | waist to hip ratio | 403689 | -2.058 | 0.727 | 0.005 | -1.642 | 0.752 | 0.029 |
| health- and fitness-related | **age at first sexual intercourse*** | 354311 | 5.632 | 0.810 | 3.58E-12 | 7.067 | 0.809 | 2.39E-18 |
| health- and fitness-related | **fluid intelligence*** | 145658 | -4.808 | 1.219 | 7.99E-05 | -5.401 | 1.273 | 2.22E-05 |
| health- and fitness-related | neuroticism score | 327994 | -0.137 | 0.830 | 0.869 | 0.166 | 0.847 | 0.845 |
| **Binary Outcomes (logistic regression)** | | | | | | | | |
| Sociodemographic | breastfed as infant | 305904 | -2.651 | 2.029 | 0.191 | -3.100 | 1.999 | 0.121 |
| Sociodemographic | college degree | 404518 | -1.471 | 1.648 | 0.372 | 0.984 | 1.668 | 0.555 |
| Sociodemographic | live in urban area | 400629 | -0.299 | 2.051 | 0.884 | -4.408 | 2.096 | 0.035 |
| Sociodemographic | **religious group attendance*** | 404518 | 9.367 | 1.778 | 1.38E-07 | 17.113 | 1.943 | 1.28E-18 |
| health- and fitness-related | diagnosed with diabetes | 403387 | -6.988 | 2.818 | 0.013 | -2.730 | 3.411 | 0.424 |
| health- and fitness-related | ever drink***** | 403990 | 5.684 | 3.498 | 0.104 | 12.333 | 3.726 | 0.001 |
| health- and fitness-related | ever smoke | 365395 | 1.648 | 1.661 | 0.321 | 3.671 | 1.670 | 0.028 |
| health- and fitness-related | Probable BPD diagnosis | 71007 | -8.191 | 10.196 | 0.422 | 1.679 | 15.005 | 0.911 |
| health- and fitness-related | Probable MDD diagnosis | 95481 | 1.389 | 3.394 | 0.682 | 1.983 | 3.655 | 0.587 |
