# Supplementary material for: Relationships between estimated autozygosity and complex traits in the UK Biobank
Source: PLoS Genet. 2018 Jul 27;14(7):e1007556. doi: 10.1371/journal.pgen.1007556 (PMC6082573; doi:10.1371/journal.pgen.1007556)
Supplement: S7 Table — All models controlled for age, age2, sex, batch number, per-sample SNP missingness, and the first 20 principal components. Phenotypes with a significant association (p < 0.002 after multiple testing correction) with FROH_long are bolded, while those with a significant relationship with FROH_short are starred. The quantitative traits (analyzed via linear regression) are listed first in the table, followed by diagnoses and binary traits (analyzed via logistic regression models). BP, blood pressure; FEV1, forced expiratory volume in 1 second; FVC, forced vital capacity; BPD, bipolar disorder; MDD, major depressive disorder; df, degrees of freedom; SE, standard error. (DOCX) [file pgen.1007556.s008.docx]

|  |  |  | **Recent Inbreeding (*Froh* from ROHs > 8.5 Mb)** | | | **Distant Inbreeding (*Froh* from ROHs < 8.5 Mb)** | | |
| --- | --- | --- | --- | --- | --- | --- | --- | --- |
| **Category** | **Trait** | **df** | **Beta** | **SE** | **p** | **Beta** | **SE** | **p** |
| **Quantitative Traits (linear regression)** | | | | | | | | |
| Sociodemographic | **income** | 347883 | -4.987 | 0.860 | 6.73E-09 | 1.734 | 0.868 | 0.046 |
| Sociodemographic | years of education | 400383 | -0.699 | 0.822 | 0.395 | -0.108 | 0.847 | 0.899 |
| Sociodemographic | Townsend Deprivation Index | 404034 | -2.550 | 0.830 | 0.002 | 1.462 | 0.858 | 0.089 |
| biometric | basal metabolic rate | 397363 | -0.353 | 0.833 | 0.672 | -1.515 | 0.858 | 0.077 |
| biometric | **birth weight** | 229569 | -3.653 | 1.124 | 0.001 | 1.916 | 1.158 | 0.098 |
| biometric | body mass index | 403173 | -1.984 | 0.837 | 0.018 | 1.117 | 0.866 | 0.197 |
| biometric | body fat percentage | 397148 | -2.074 | 0.835 | 0.013 | 1.060 | 0.860 | 0.218 |
| biometric | diastolic BP | 380686 | 2.277 | 0.862 | 0.008 | -0.617 | 0.892 | 0.489 |
| biometric | systolic BP | 379733 | 2.215 | 0.824 | 0.007 | -0.755 | 0.851 | 0.375 |
| biometric | **forced expiratory volume in 1 second (FEV1)** | 304301 | -3.328 | 0.890 | 1.83E-04 | -2.031 | 0.886 | 0.022 |
| biometric | FEV1/FVC | 304301 | -1.817 | 0.985 | 0.065 | 0.641 | 0.981 | 0.514 |
| biometric | **height** | 403609 | -3.010 | 0.817 | 2.30E-04 | -0.576 | 0.845 | 0.496 |
| biometric | grip strength | 403589 | -2.315 | 0.801 | 0.004 | -1.071 | 0.828 | 0.196 |
| biometric | waist to hip ratio | 403689 | -1.684 | 0.825 | 0.041 | -0.820 | 0.853 | 0.336 |
| health- and fitness-related | **age at first sexual intercourse*** | 354311 | 3.025 | 0.911 | 0.001 | 5.683 | 0.910 | 4.20E-10 |
| health- and fitness-related | fluid intelligence | 145658 | -3.041 | 1.384 | 0.028 | -3.895 | 1.446 | 0.007 |
| health- and fitness-related | neuroticism score | 327994 | -0.271 | 0.937 | 0.773 | 0.295 | 0.957 | 0.758 |
| **Binary Outcomes (logistic regression)** | | | | | | | | |
| Sociodemographic | breastfed as infant | 305904 | -1.523 | 2.291 | 0.506 | -2.415 | 2.251 | 0.283 |
| Sociodemographic | college degree | 404518 | -2.465 | 1.868 | 0.187 | 2.165 | 1.889 | 0.252 |
| Sociodemographic | live in urban area | 400629 | 2.250 | 2.360 | 0.340 | -5.526 | 2.394 | 0.021 |
| Sociodemographic | religious group attendance* | 404518 | 2.046 | 2.107 | 0.331 | 16.008 | 2.255 | 1.27E-12 |
| health- and fitness-related | diagnosed with diabetes | 403387 | -7.651 | 3.274 | 0.019 | 1.541 | 3.935 | 0.695 |
| health- and fitness-related | ever drink | 403990 | -0.198 | 4.247 | 0.963 | 12.441 | 4.383 | 0.005 |
| health- and fitness-related | ever smoke | 365395 | -0.083 | 1.885 | 0.965 | 3.710 | 1.891 | 0.050 |
| health- and fitness-related | probable BPD diagnosis | 71007 | -11.664 | 11.840 | 0.325 | 9.035 | 16.970 | 0.594 |
| health- and fitness-related | probable MDD diagnosis | 95481 | 0.676 | 3.831 | 0.860 | 1.641 | 4.136 | 0.691 |
