# Supplementary material for: Relationships between estimated autozygosity and complex traits in the UK Biobank
Source: PLoS Genet. 2018 Jul 27;14(7):e1007556. doi: 10.1371/journal.pgen.1007556 (PMC6082573; doi:10.1371/journal.pgen.1007556)
Supplement: S9 Table — Phenotypes with a significant relationship with FROH (p < 0.003 after multiple testing correction) are bolded, while those with a significant relationship with FSNP are italicized. The quantitative traits (analyzed via linear regression) are listed first in the table, followed by diagnoses and binary traits (analyzed via logistic regression models). BP, blood pressure; FEV1, forced expiratory volume in 1 second; FVC, forced vital capacity; BPD, bipolar disorder; MDD, major depressive disorder; df, degrees of freedom; SE, standard error. (DOCX) [file pgen.1007556.s010.docx]

|  |  | ***F_ROH_ Regression Coefficient*** | | | ***F_SNP_ Regression Coefficient*** | | |
| --- | --- | --- | --- | --- | --- | --- | --- |
| **Category** | **Trait** | **Beta*_FROH_*** | **SE*_FROH_*** | ***p_FROH_*** | **Beta*_FSNP_*** | **SE*_FSNP_*** | ***p_FSNP_*** |
| **Quantitative Traits (linear regression)** | | | | | | | |
| biometric | basal metabolic rate | -0.898 | 0.781 | 0.250 | 0.301 | 0.392 | 0.443 |
| biometric | birth weight | -1.645 | 0.925 | 0.075 | 0.440 | 0.464 | 0.343 |
| biometric | body mass index | -0.212 | 0.774 | 0.784 | -0.255 | 0.389 | 0.512 |
| biometric | body fat percentage | -0.271 | 0.774 | 0.726 | 0.084 | 0.389 | 0.829 |
| biometric | diastolic BP | 1.608 | 0.806 | 0.046 | -0.245 | 0.405 | 0.545 |
| biometric | systolic BP | 1.002 | 0.759 | 0.186 | -0.513 | 0.381 | 0.178 |
| biometric | forced expiratory volume in 1 second | -1.571 | 0.797 | 0.049 | -0.859 | 0.393 | 0.029 |
| biometric | FEV1/FVC | -0.066 | 0.876 | 0.940 | 0.356 | 0.432 | 0.410 |
| biometric | height | -0.977 | 0.755 | 0.196 | -0.294 | 0.380 | 0.438 |
| biometric | grip strength | -0.408 | 0.747 | 0.585 | -0.841 | 0.372 | 0.024 |
| biometric | *waist to hip ratio* | 0.120 | 0.759 | 0.875 | -1.590 | 0.382 | 3.11E-05 |
| health- and fitness-related | **age at first sexual intercourse** | 2.432 | 0.780 | 0.002 | 0.624 | 0.384 | 0.104 |
| health- and fitness-related | fluid intelligence | -3.061 | 1.142 | 0.007 | -0.319 | 0.588 | 0.588 |
| health- and fitness-related | neuroticism score | -0.031 | 0.841 | 0.970 | -0.137 | 0.423 | 0.746 |
| **Binary Outcomes (logistic regression)** | | | | | | | |
| health- and fitness-related | diagnosed with diabetes | 0.769 | 3.754 | 0.838 | -0.617 | 1.993 | 0.757 |
| health- and fitness-related | ever drink | -0.159 | 4.482 | 0.972 | 1.186 | 2.470 | 0.631 |
| health- and fitness-related | ever smoke | -0.607 | 1.739 | 0.727 | 2.030 | 0.867 | 0.019 |
| health- and fitness-related | probable BPD diagnosis | 8.155 | 18.002 | 0.651 | 4.643 | 7.045 | 0.510 |
| health- and fitness-related | probable MDD diagnosis | -0.015 | 3.392 | 0.996 | 0.152 | 1.796 | 0.933 |
