# Supplementary figures and images for: Relationships between estimated autozygosity and complex traits in the UK Biobank
Source: PLoS Genet. 2018 Jul 27;14(7):e1007556. doi: 10.1371/journal.pgen.1007556 (PMC6082573; doi:10.1371/journal.pgen.1007556)

**
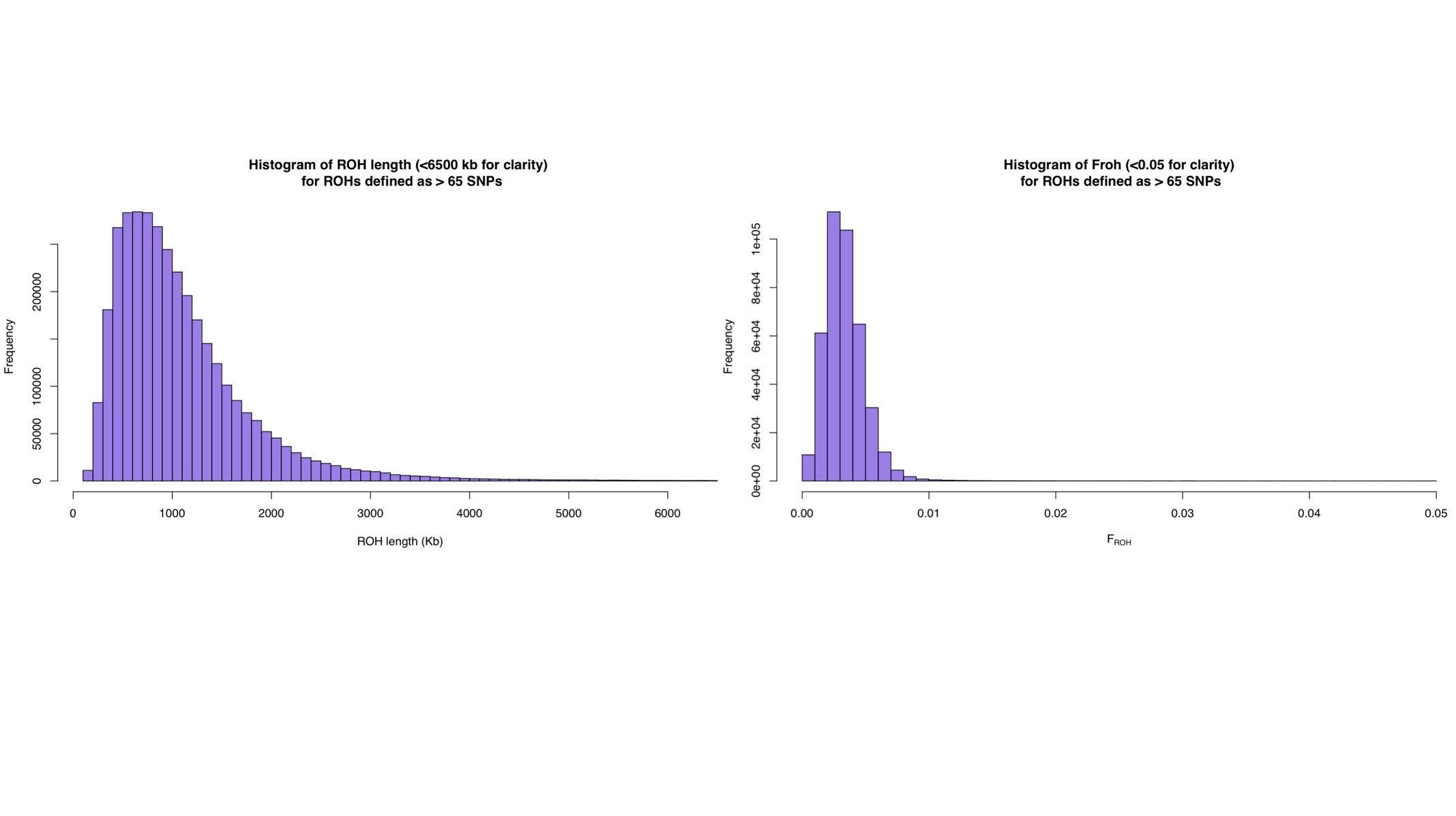
**

Supplement: S1 Fig — The histogram of ROH lengths is cut off at 6500 Kb for clarity; similarly, the histogram of FROH is cut off at 0.05 for clarity. There were 583 individuals who had FROH > 0.05; these individuals were not excluded from analyses. (DOCX) [file pgen.1007556.s011.docx]
