# Supplementary material for: Relationships between estimated autozygosity and complex traits in the UK Biobank
Source: PLoS Genet. 2018 Jul 27;14(7):e1007556. doi: 10.1371/journal.pgen.1007556 (PMC6082573; doi:10.1371/journal.pgen.1007556)
Supplement: Supplementary file 12 [file pgen.1007556.s012.docx]

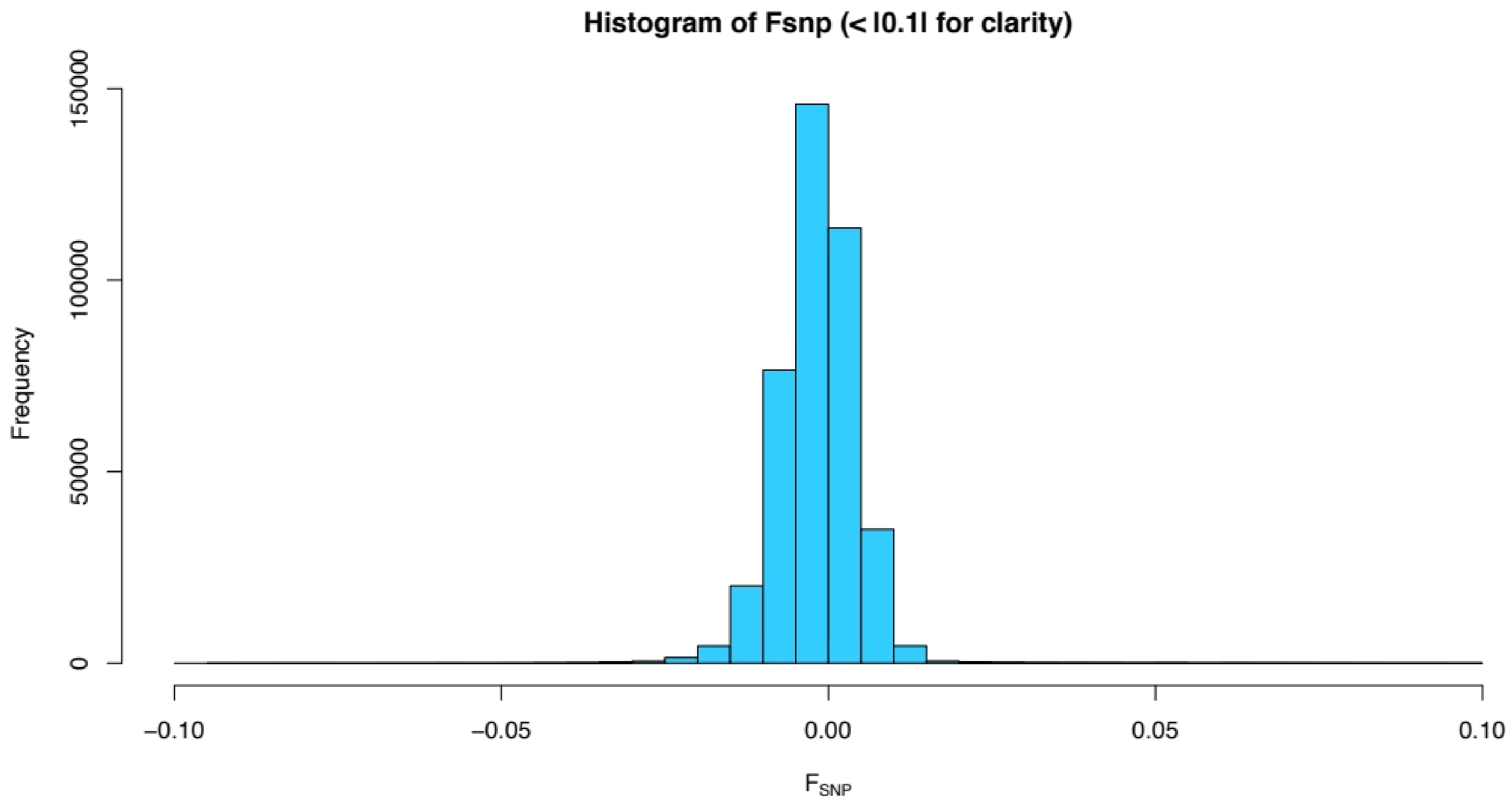

Histogram of Fsnp (< |0.1| for clarity)
Frequency
0
50000
100000
150000
−0.10
−0.05
0.00
0.05
0.10
F_{SNP}
